# Macrophages inhibit *Coxiella burnetii* by the ACOD1‐itaconate pathway for containment of Q fever

**DOI:** 10.15252/emmm.202215931

**Published:** 2022-12-07

**Authors:** Lisa Kohl, Md Nur A Alam Siddique, Barbara Bodendorfer, Raffaela Berger, Annica Preikschat, Christoph Daniel, Martha Ölke, Elisabeth Liebler‐Tenorio, Jan Schulze‐Luehrmann, Michael Mauermeir, Kai‐Ting Yang, Inaya Hayek, Manuela Szperlinski, Jennifer Andrack, Ulrike Schleicher, Aline Bozec, Gerhard Krönke, Peter J Murray, Stefan Wirtz, Masahiro Yamamoto, Valentin Schatz, Jonathan Jantsch, Peter Oefner, Daniel Degrandi, Klaus Pfeffer, Katja Mertens‐Scholz, Simon Rauber, Christian Bogdan, Katja Dettmer, Anja Lührmann, Roland Lang

**Affiliations:** ^1^ Mikrobiologisches Institut – Klinische Mikrobiologie, Immunologie und Hygiene Universitätsklinikum Erlangen, Friedrich‐Alexander‐Universität (FAU) Erlangen‐Nürnberg Erlangen Germany; ^2^ Institute of Functional Genomics University of Regensburg Regensburg Germany; ^3^ Department of Nephropathology Universitätsklinikum Erlangen, Friedrich‐Alexander‐Universität Erlangen‐Nürnberg Erlangen Germany; ^4^ Institute of Molecular Pathogenesis, Friedrich‐Loeffler‐Institut, Federal Research Institute for Animal Health Jena Germany; ^5^ Department of Medicine 3 Universitätsklinikum Erlangen, Friedrich‐Alexander‐Universität Erlangen‐Nürnberg Erlangen Germany; ^6^ Deutsches Zentrum für Immuntherapie (DZI) Friedrich‐Alexander‐Universität Erlangen‐Nürnberg and Universitätsklinikum Erlangen Erlangen Germany; ^7^ Institute of Bacterial Infections and Zoonoses, Friedrich‐Loeffler‐Institut, Federal Research Institute for Animal Health Jena Germany; ^8^ Medical Immunology Campus Erlangen FAU Erlangen‐Nürnberg Erlangen Germany; ^9^ Max Planck Institute for Biochemistry Martinsried Germany; ^10^ Department of Medicine 1 Universitätsklinikum Erlangen, Friedrich‐Alexander‐Universität Erlangen‐Nürnberg Erlangen Germany; ^11^ Immunology Frontier Research Center Osaka University Osaka Japan; ^12^ Institute of Clinical Microbiology University Hospital Regensburg Regensburg Germany; ^13^ Institute of Medical Microbiology Heinrich Heine University Düsseldorf Düsseldorf Germany; ^14^ Present address: Institute for Medical Microbiology, Immunology and Hygiene University Hospital Cologne and Faculty of Medicine, University of Cologne Cologne Germany

**Keywords:** Cis‐aconitate decarboxylase 1, *Coxiella burnetii*, Immune responsive gene 1, immunometabolism, itaconate, Immunology, Microbiology, Virology & Host Pathogen Interaction

## Abstract

Infection with the intracellular bacterium *Coxiella (C.) burnetii* can cause chronic Q fever with severe complications and limited treatment options. Here, we identify the enzyme cis‐aconitate decarboxylase 1 (ACOD1 or IRG1) and its product itaconate as protective host immune pathway in Q fever. Infection of mice with *C. burnetii* induced expression of several anti‐microbial candidate genes, including *Acod1*. In macrophages, *Acod1* was essential for restricting *C. burnetii* replication, while other antimicrobial pathways were dispensable. Intratracheal or intraperitoneal infection of *Acod1* ^−/−^ mice caused increased *C. burnetii* burden, weight loss and stronger inflammatory gene expression. Exogenously added itaconate restored pathogen control in *Acod1* ^−/−^ mouse macrophages and blocked replication in human macrophages. In axenic cultures, itaconate directly inhibited growth of *C. burnetii*. Finally, treatment of infected *Acod1* ^−/−^ mice with itaconate efficiently reduced the tissue pathogen load. Thus, ACOD1‐derived itaconate is a key factor in the macrophage‐mediated defense against *C. burnetii* and may be exploited for novel therapeutic approaches in chronic Q fever.

## Graphical abstract

The intracellular bacterium *Coxiella burnetii* causes the anthropozoonotic infection Q fever. Most patients control and resolve infection, but some develop chronic disease. Finding out how macrophages inhibit bacterial replication may explain susceptibility and suggest new therapies.

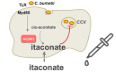

## Introduction

Q fever is an anthropozoonotic infection caused by *Coxiella (C.) burnetii*, mostly acquired by inhalation of infectious aerosol released from birth products of infected goats, sheep or cattle. Acute Q fever is characterized primarily by respiratory symptoms and pneumonia, but can also manifest as hepatitis (Eldin *et al*, 2017). In most patients, the disease is self‐limiting and inflammation resolves within a few weeks. However, 1–5% of patients will develop chronic Q fever (Kampschreur *et al*, 2014), with persistent replication of *C. burnetii* in various tissues, including the vascular system. Severe complications such as endocarditis and mycotic aneurysms can lead to Q fever‐related death in 25% of patients (van Roeden *et al*, 2019). Chronic Q fever is difficult to treat with antibiotics such as tetracyclines and fluoroquinolones, which show limited efficacy even after extended courses of 18–24 months.

*Coxiella burnetii* is an intracellular bacterium that resists the harsh environment of the macrophage phagolysosome and in fact requires an acidic pH for replication. The bacterium expresses a type IV secretion system (T4SS) for injection of effector proteins into the host cell cytoplasm, which is required for formation of the *Coxiella‐*containing vacuole (CCV) and intracellular replication (Carey *et al*, 2011; Lührmann *et al*, 2017). Suppression of phagosome acidification by the drug hydroxychloroquine synergizes with the antibiotic doxycycline *in vitro* (Raoult *et al*, 1990). The combination of hydroxychloroquine with doxycycline is the currently recommended medical treatment for chronic Q fever (Anderson *et al*, 2013).

Studies in mouse models showed that immunologic control of *C. burnetii* requires CD4 and CD8 T lymphocytes and production of IFNγ (Andoh *et al*, 2007), the essential cytokine for arming macrophages to combat intracellular bacteria. Innate immune cells detect *C. burnetii* through Toll‐like receptors (TLR) and MyD88‐signaling (Zamboni *et al*, 2004; Ammerdorffer *et al*, 2015; Ramstead *et al*, 2016; Kohl *et al*, 2019). The effector mechanisms induced by IFNγ and MyD88 signaling to enable macrophages for the killing of *C. burnetii* are incompletely understood. In the past, a protective role has been demonstrated for the inducible or type 2 nitric oxide (NO) synthase (iNOS or NOS2) (Howe *et al*, 2002; Zamboni & Rabinovitch, 2003) that is strongly induced by combined TLR and IFNγ activation and generates NO from the amino acid L‐arginine (Bogdan, 2015).

Among the large number of genes induced by IFNγ in myeloid cells, several other important antimicrobial proteins have been identified that mediate defense against various intracellular pathogens. These include 47 kDa and 65 kDa guanylate‐binding proteins (GBPs) that are recruited to phagosomes containing *Mycobacterium (M.) tuberculosis* (Kim *et al*, 2011) and *Toxoplasma (T.) gondii* (Yamamoto *et al*, 2012; Degrandi *et al*, 2013; Kravets *et al*, 2016). Another example is the tryptophan‐depleting enzyme indoleamine‐2,3‐deoxygenase (IDO) 1 which inhibits *Francisella (F.) tularensis* replication in the lung (Peng & Monack, 2010). While the function of GBP family members in infection with *C. burnetii* has not yet been addressed, a recent study reported that IDO1 impairs growth of *C. burnetii* in human THP1 macrophages (Ganesan & Roy, 2019).

During the past 10 years, a series of studies highlighted the importance of metabolic pathways in the host inflammatory response to infection (O'Neill & Pearce, 2016). Macrophages respond to TLR activation with a glycolytic switch (the so‐called Warburg effect) and alterations in the tricarboxylic acid (TCA) cycle that include accumulation of succinate (Ryan & O'Neill, 2020). These changes in central energy metabolism of the cell also impact on the transcriptional regulation of gene expression in response to microbial danger, including enhanced expression of IL‐1 (Ryan & O'Neill, 2020). The TCA cycle metabolite citrate plays an important role in immunity because it is essential for fatty acid synthesis and, in addition, can be metabolized to itaconate (ITA) that has immunoregulatory and anti‐microbial functions (Williams & O'Neill, 2018). Citrate supports replication of *C. burnetii* and is an essential component of the axenic growth medium of *C. burnetii* (Omsland *et al*, 2009). In macrophages infected with *C. burnetii*, high intracellular citrate levels induced by the transcription factor STAT3 promote bacterial replication, whereas hypoxia inhibits STAT3 activation, citrate accumulation and *C. burnetii* growth through HIF1α (Hayek *et al*, 2019).

ITA is derived from cis‐aconitate, which is an intermediate in the conversion of citrate to isocitrate within the TCA cycle and serves as a substrate for the enzyme cis‐aconitate decarboxylase (ACOD1) (encoded by *Acod1*, also termed immune responsive gene‐1 [*Irg1*]). ACOD1 is associated with mitochondria (Degrandi *et al*, 2009) and catalyzes the decarboxylation of cis‐aconitate to ITA (Michelucci *et al*, 2013). ITA has recently received a lot of attention for its immunoregulatory effects (Mills *et al*, 2018), but it can also inhibit the growth of several bacteria in axenic culture including *M. tuberculosis* (Michelucci *et al*, 2013) and *Legionella (L.) pneumophila* (Naujoks *et al*, 2016). Following infection with *M. tuberculosis*, *Acod1* ^−/−^ mice developed higher bacterial burden and dysregulated neutrophil accumulation in the lung, but no direct effect on intracellular growth was found in *Acod1* ^−/−^ macrophages (Nair *et al*, 2018). More recently, the intracellular growth of *M. avium* (Gidon *et al*, 2021) and *Brucella species* (Lacey *et al*, 2021) was reported to be inhibited by the ACOD1‐ITA axis. In addition, ACOD1 also contributes to restriction of Zika virus infection in the CNS (Daniels *et al*, 2019).

In a recently established mouse model of infection with *C. burnetii* Nine Mile Phase II (NMII), we observed that efficient clearance of NMII from spleen, liver and lung was dependent on the TLR adapter protein MyD88 (Kohl *et al*, 2019). The increased bacterial burden seen in MyD88‐deficient mice was accompanied by reduced expression of pro‐inflammatory genes like CCL2 and IFNγ and less leukocyte infiltration into the tissues. Here, we identify the ACOD1‐ITA axis as a key MyD88‐dependent macrophage‐autonomous determinant of early containment of *C. burnetii* and control of Q fever.

## Results

### ACOD1 belongs to a group of MyD88‐dependent genes upregulated by *C. burnetii* infection *in vivo*

**Figure 1:**
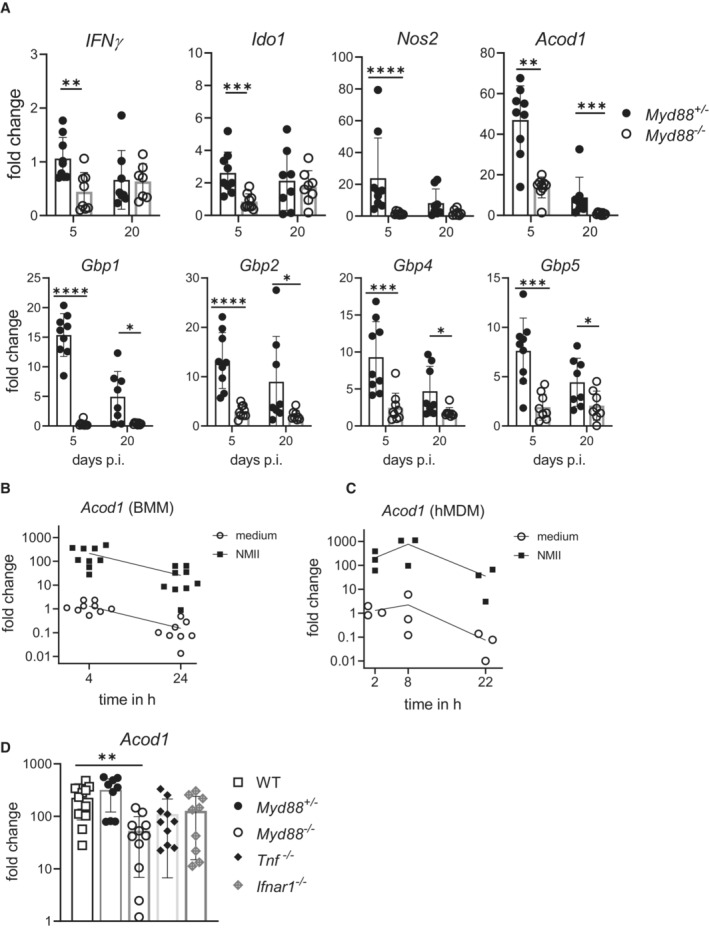
ACOD1 belongs to a group of MyD88‐dependent genes upregulated by *Coxiella burnetii* infection *in vivo* and *in vitro* *Myd88* ^+/−^ and *Myd88* ^−/−^ mice were infected i.p. with NMII. Spleen RNA was prepared on day 5 or day 20 post‐infection. Expression of *Nos2*, *Gbp1*, *Gbp2*, *Gbp5*, *Ido1* and *Acod1* was analyzed by qRT‐PCR, using non‐infected *Myd88* ^+/−^ as calibrator. Mean and SD, *n* = 8–9 mice per genotype, pooled from three experiments. Data for expression of *IFNγ*, *Nos2* and *Gbp1* on day 5 were previously reported (Kohl *et al*, 2019). Mann–Whitney test was performed between genotypes of indicated time points. *P < 0.05, **P < 0.01, ***P < 0.001, ****P < 0.0001.Induction of *Acod1* mRNA in mouse BMM infected with MOI 10 of NMII for 4 and 24 h (fold change relative to mock controls, *n* = 9 mice pooled from five experiments, data points staggered).
*Acod1* mRNA expression in human GM‐CSF‐driven monocyte‐derived macrophages (MDM) infected with MOI 30 for 4 h and harvested at the indicated times, *n* = 3 different donors, each data point represents average value of triplicate stimulations.
*Acod1* expression in BMM is partially dependent on MyD88 but not on *Tnf* and *Ifnar1*. BMM were infected with NMII (MOI 10) for 4 h. *Acod1* mRNA shown as fold induction relative to non‐infected WT controls. Each dot represents one mouse (*n* = 9–13 mice), which were pooled from three to five independent experiments. Bars show mean with SD. One‐way ANOVA with Dunnett's multiple comparisons test was performed. **P < 0.01.

To dissect the mechanisms of MyD88‐dependent killing of *C. burnetii*, we explored the expression of several genes with known or assumed anti‐microbial function in the spleen of mice after intraperitoneal infection with NMII. Consistent with reduced IFNγ expression, the mRNA induction of *Nos2*, *Gbp1*, *Gbp2*, *Gbp4*, *Gbp5*, *Ido1* and *Acod1* was severely impaired in the spleens of *Myd88* ^−/−^ mice after intraperitoneal infection (Fig 1A). Infection of primary murine and human macrophages *in vitro* rapidly triggered expression of *Acod1* (Fig 1B and C). As *in vivo*, the MyD88 pathway was required for robust induction of *Acod1* in murine BMM, whereas TNF and type 1 IFN signaling, which both can enhance *Acod1* expression (Shi *et al*, 2005; Gidon *et al*, 2021), was less essential (Fig 1D).

### ACOD1‐deficient macrophages allow *C. burnetii* replication *in vitro*

**Figure 2:**
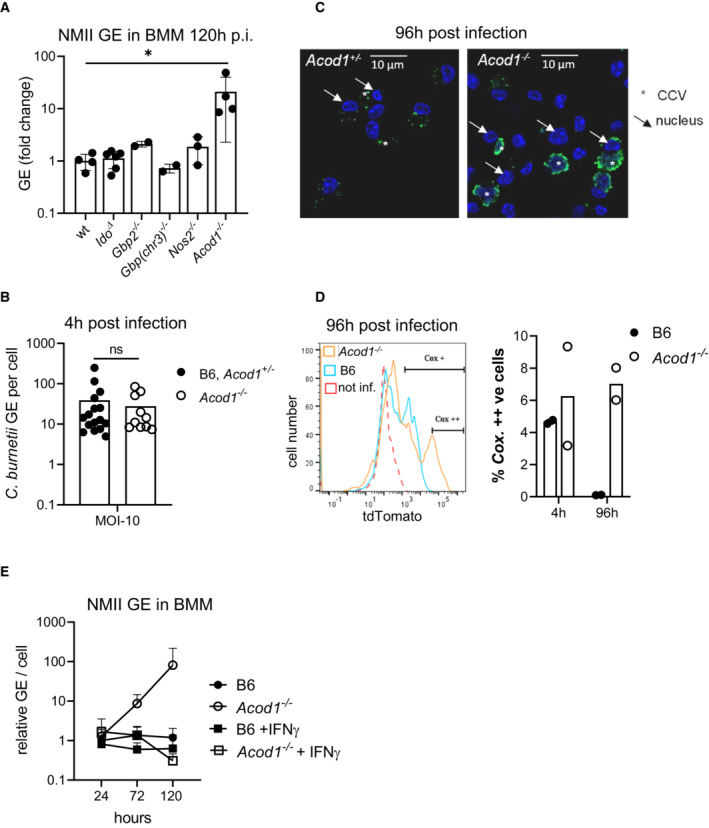
ACOD1‐deficient macrophages allow *Coxiella burnetii* replication *in vitro* BMM of the indicated genotypes were infected with NMII (MOI 10) for 4 h, followed by removal of extracellular bacteria by gentle washing. Genome equivalents (GE) of NMII per cell were measured by qPCR after 120 h in macrophage lysates. Average GE values from duplicate wells were normalized to the mean GE of all WT control samples. Each dot represents BMM from one mouse. Bars show mean with SD. *P < 0.05 in Kruskal–Wallis test with Dunn's multiple comparisons test.Uptake of *C. burnetii* during initial infection period is comparable in *Acod1* ^−/−^ and control BMM. GE per cell were determined after 4 h of incubation with NMII (MOI 10) and removal of extracellular bacteria. Each dot represents BMM from one mouse. Pooled from 4–6 experiments. ns not significant in Mann–Whitney test.Immunofluorescence microscopy of WT and *Acod1* ^−/−^ BMM 96 h after infection. Staining for *C. burnetii* appears in green, DAPI stain for DNA shows nuclei (examples marked by arrows) and large CCV in *Acod1* ^−/−^ BMM (marked by asterisk).Flow cytometric detection of tdTomato‐expressing NMII in BMM 4 and 96 h after infection. Representative histogram overlay (after 96 h) (red dotted line: WT, LPS; blue: WT, infected; orange: *Acod1* ^−/−^, infected) and quantitation of highly tdTomato‐positive BMM from 2 independent experiments, each dot represents averages of duplicates from one mouse.Treatment with IFNγ overcomes the defect of ACOD1‐deficient BMM to control NMII replication. Average GE values from duplicate or triplicate wells were normalized to the mean GE of B6 control samples at 24 h to obtain fold change values. Mean and SD of *n* = 5 mice pooled from two independent experiments.

We next employed bone‐marrow‐derived macrophages (BMM) deficient in these candidate anti‐microbial effector genes to test their requirement for control of NMII replication *in vitro* (Fig 2A). A previous study reported that the production of NO by infected macrophages reduces the size of CCVs and thereby the intracellular load of *C. burnetii* (Zamboni & Rabinovitch, 2003). Using *Nos2* ^−/−^ macrophages, we observed only a weak, non‐significant increase in the NMII burden when compared with wild‐type controls (Fig 2A). Several members of the guanylate‐binding protein (GBP) family of IFNγ‐inducible 65 kDa GTPases are recruited to the phagosomal/endosomal membrane in macrophages during infection with intracellular bacteria or protozoa and can cooperate to destroy intravacuolar pathogens. However, macrophages deficient in *Gbp2* or with a combined deletion of *Gbp1*, *Gbp2*, *Gbp3*, *Gbp5* and *Gbp7* encoded on chromosome 3 still efficiently inhibited NMII replication (Fig 2A). IDO1 is the rate‐limiting enzyme in tryptophan catabolism and has immunoregulatory as well as antimicrobial effects. Since tryptophan is an essential amino acid for *C. burnetii* (Sandoz *et al*, 2016), its intracellular depletion by IDO1 might constitute a nutritional control mechanism of macrophages as was recently shown in human macrophages (Ganesan & Roy, 2019). However, BMM harboring a combined deletion of *Ido1* and *Ido2* did not show any defect in restricting NMII replication (Fig 2A). In contrast, macrophages from *Acod1* ^−/−^ mice were unable to restrict intracellular replication of NMII and harbored at least 10‐fold more *Coxiella* after 120 h (Fig 2A), similar to *Myd88* ^−/−^ macrophages (Kohl *et al*, 2019). Initial infection levels after 4 h were not different between *Acod1* ^−/−^ and control BMM, indicating that phagocytosis of *C. burnetii* was not affected (Fig 2B).

**Figure EV1:**
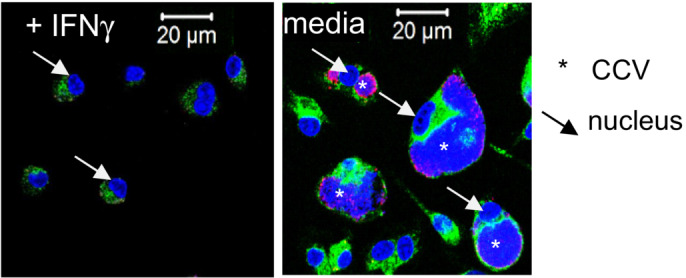
Immunofluorescence microscopy of *Acod1* ^−/−^ BMM treated with IFNγ Immunofluorescence microscopy of *Acod1* ^−/−^ BMM 120 h after infection with NMII at MOI 10. IFNγ was added or not 4 h after infection when extracellular *Coxiella burnetii* was removed. Staining for Lamp1 appears in green, staining for *C. burnetii* in pink, DAPI stain for DNA shows nuclei (examples marked by arrows) and large CCV in *Acod1* ^−/−^ BMM in the absence of IFNγ (marked by asterisk).

To validate the strong effect of ACOD1‐deficiency on the abundance of *C. burnetii* genome equivalents detected by qPCR, we employed immunofluorescence staining. While *Acod1* ^+/−^ macrophages showed only discrete staining with an anti‐*C. burnetii* antiserum, large CCV were observed in *Acod1* ^−/−^ macrophages (Fig 2C). Flow cytometry of macrophages infected with tdTomato‐expressing NMII confirmed this effect in the absence of ACOD1, with around 6% of infected cells displaying a very strong fluorescent signal (Fig 2D), corresponding to strongly enlarged CCVs filled with bacteria seen in Fig 2C. While NMII strongly replicated in *Acod1* ^−/−^ BMM, treatment with IFNγ starting 4 h after the infection, when extracellular NMII were washed away, inhibited bacterial replication in both WT and *Acod1* ^−/−^ BMM as shown by qPCR for *C. burnetii* GE (Fig 2E) and immunofluorescence staining (Fig EV1). These data demonstrate that ACOD1 is essential for the cell‐autonomous, but not for the IFNγ‐driven control of *C. burnetii* growth.

### ACOD1‐deficient mice have increased bacterial burden after infection with *C. burnetii*

**Figure 3:**
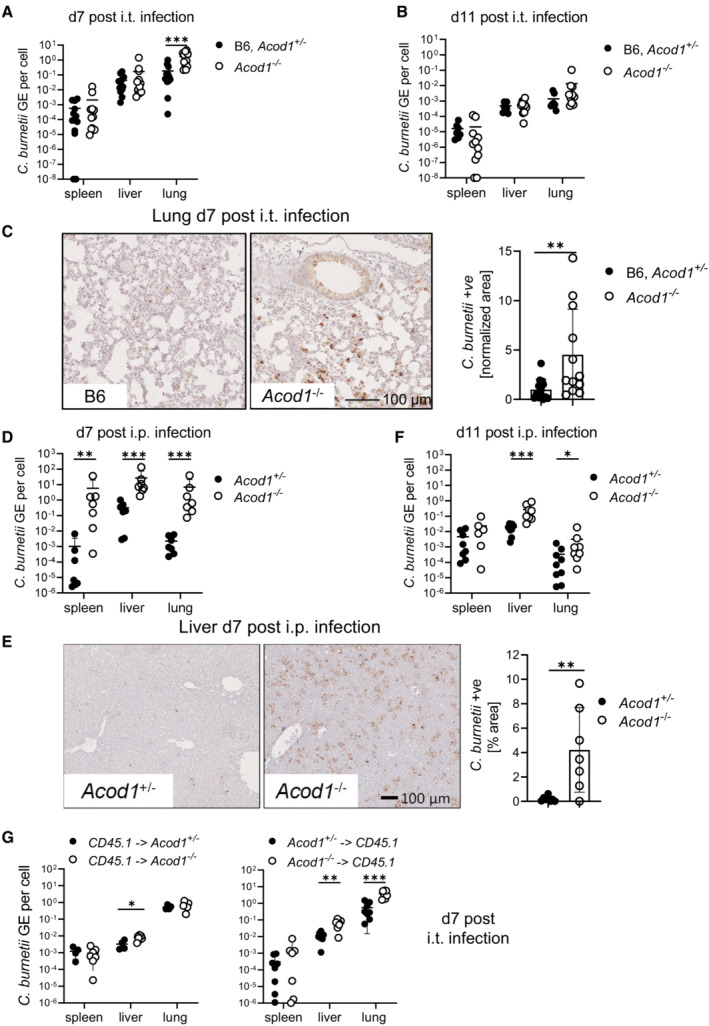
Increased bacterial burden in ACOD1‐deficient mice infected with *Coxiella burnetii* AMice were infected by the i.t. route with 10^6^ NMII. Bacterial load in lung, spleen and liver on d7 after i.t. infection. *n* = 12 for *Acod1* ^−/−^, *n* = 13 for B6, *Acod1* ^+/−^ (pooled from three independent experiments).BBacterial load on d11 after i.t. infection. *n* = 10–11 for *Acod1* ^−/−^, *n* = 8 for B6, *Acod1* ^+/−^ (pooled from two independent experiments).C–F(C) Immunohistochemistry for *C. burnetii* in the lung on d7 after i.t. infection. Representative images and quantitation of lung tissue positive for *C. burnetii* (*n* = 12–13 mice, pooled from three experiments, each normalized to the mean of WT or *Acod1* ^+/−^ infected mice). Each dot represents one mouse. Bars show mean and SD. (D, F) Bacterial load in lung, spleen and liver on d7 (D) and d11 (F) after i.p. infection with 5 × 10^7^ NMII. *n* = 6–9 mice per genotype, pooled from two independent experiments. (E) Immunohistochemistry for *C. burnetii* on day 7 after intraperitoneal infection. Each dot represents one mouse. Bars show mean with SD.GRadiation chimeric mice were infected i.t. with NMII. Each dot represents one mouse, data are pooled from two experiments. Bacterial burden in the tissues was determined after 7 days, except for one experiment with CD45.1 recipients when mice were sacrificed after 5 days because of weight loss. *n* = 4–8 mice. Data information: Statistical analyses comparing *Acod1* ^−/−^ to B6 or *Acod1* ^+/−^ controls with Mann–Whitney test, *P < 0.05, **P < 0.01, ***P < 0.001.

We next asked whether ACOD1 was required for control of *C. burnetii* during infection *in vivo*. We infected mice by the intratracheal route with NMII. Seven days after infection, the bacterial burden in the lungs of *Acod1* ^−/−^ mice was more than 10‐fold higher than in wild‐type or *Acod1* ^+/−^ animals (Fig 3A). Interestingly, this increased pulmonary bacterial replication was transient, as wild‐type and *Acod1* ^−/−^ mice had comparable NMII numbers in the lung on day 11 (Fig 3B). Immunohistochemistry for *C. burnetii* in the lungs confirmed a strongly increased bacterial load in *Acod1* ^−/−^ mice on day 7 after i.t. infection (Fig 3C). As the NMII load in spleen and liver was not significantly different between the genotypes (Fig 3A and B), we wondered whether ACOD1 was exclusively required for early control of *C. burnetii* in the lung in a tissue‐specific manner, or whether the route of infection played a role. Infection of *Acod1* ^−/−^ and littermate *Acod1* ^+/−^ control mice by intraperitoneal injection revealed significantly higher bacterial loads in spleen, liver and lungs of *Acod1* ^−/−^ mice on day 7 (Fig 3D), which was confirmed by immunohistochemistry of liver sections (Fig 3E), demonstrating that ACOD1 is indeed required for the control of *C. burnetii* in all organs tested. On day 11 after i.p. infection, the bacterial burden was strongly reduced in *Acod1* ^−/−^ mice compared with day 7 (Fig 3F). Although *C. burnetii* GE was still higher in the liver and lungs of *Acod1* ^−/−^ than in the *Acod1* ^+/−^ mice (Fig 3F), the differences between the genotypes were much less pronounced than on day 7 (Fig 3D). Thus, ACOD1 is particularly important for control of *C. burnetii* during the early phase after intratracheal and after intraperitoneal infection. Finally, to determine whether ACOD1 is necessary in hematopoietic or epithelial cells or in both compartments, bone marrow chimeric mice were generated and infected by intratracheal injection. NMII burden in the lungs of irradiated WT mice reconstituted with *Acod1* ^−/−^ bone marrow was robustly increased, whereas no difference was found in the lungs of *Acod1* ^−/−^ compared with *Acod1* ^+/−^ recipients of CD45.1 bone marrow cells (Fig 3G). Albeit there was a significant increase in NMII load in the livers of *Acod1* ^−/−^ recipients, also in this organ ACOD1‐deficiency in the donor had a stronger effect (Fig 3G). Together, the data from the radiation chimeras demonstrated a requirement for ACOD1 primarily in hematopoietic cells. These findings, which are consistent with the reported predominant myeloid expression of *Acod1* (Wu *et al*, 2020), further underline a cell‐autonomous effect of ACOD1 in infected macrophages.

### Increased inflammatory and anti‐microbial gene expression in ACOD1‐deficient mice after *C. burnetii* infection

**Figure 4:**
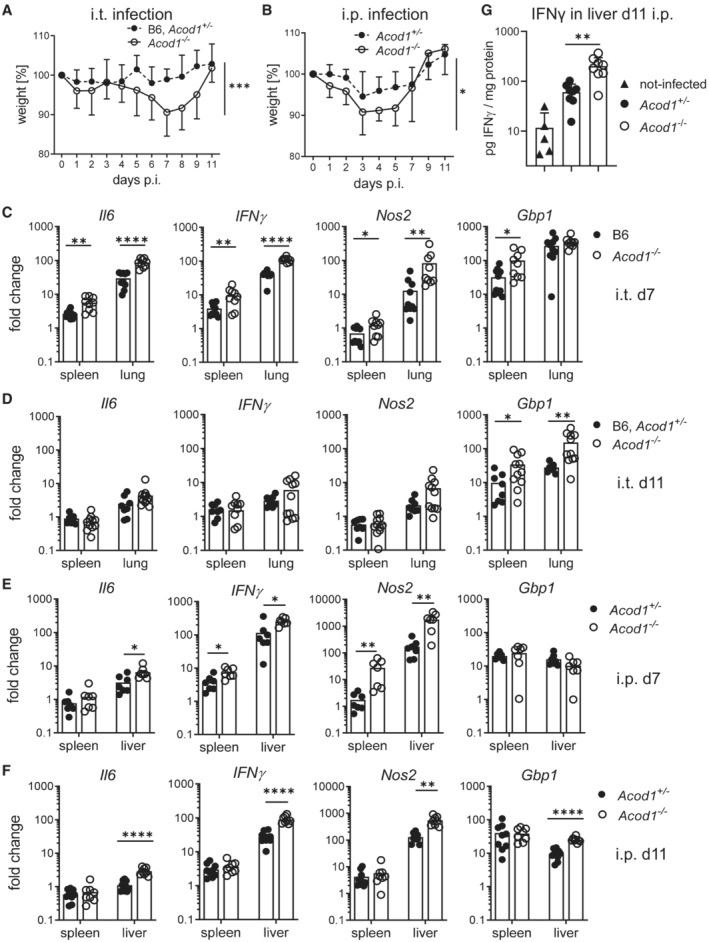
Weight loss and increased inflammatory and anti‐microbial gene expression in ACOD1‐deficient mice after *Coxiella burnetii* infection A, B(A) Weight curve until d11 after i.t. infection. Pooled data from six experiments. Mean and SD, *n* = 11–18 mice per group for days 1, 3, 8; *n* = 7–8 mice per group for days 5, 9, 11; *n* = 21–22 mice per group for days 2, 4, 6, 7. (B) Weight curve until d11 after i.p. infection. Pooled data from four experiments. Mean and SD, *n* = 7–9 mice for days 2, 3, 4, 9, 11 and *n* = 15–16 mice per genotype for days 1, 5, 7. Asterisks in (A and B) indicate *P < 0.05, ***P < 0.001 (Mixed effects model with Geisser–Greenhouse correction, comparing genotypes).C–F(C, D) Expression of *Il6*, *IFNγ*, *Nos2*, *Gbp1* in lung and spleen on d7 (C) and d11 (D) after i.t. infection. Each dot represents one mouse. *n* = 8–11, pooled from two experiments. (E, F) Gene expression changes in spleen and liver after i.p. infection on d7 (E) or d11 (F). *n* = 7–9 mice, pooled from two independent experiments. Fold changes in (C‐F) were calculated according to the ΔΔCT method, using average ΔCT values from samples of non‐infected B6 or *Acod1* ^+/−^ mice as calibrators. Asterisks in (C‐F) indicate *P < 0.05, **P < 0.01, ****P < 0.0001 (Mann–Whitney test comparing genotypes in specified organ).GProtein levels of IFNγ per mg protein in liver homogenates of non‐infected Acod1^+/−^ or B6 (*n* = 5), and *Acod1* ^−/−^ and *Acod1* ^+/−^ mice sacrificed on day 11 after i.p. infection with NMII (*n* = 8 per genotype). **P < 0.01 in Mann–Whitney test comparing infected *Acod1* ^−/−^ and *Acod1* ^+/−^.

Intratracheal infection of *Acod1* ^−/−^ mice led to a significant loss of body weight, which reached a maximum on day 7 after infection (Fig 4A). Similar to the bacterial burden in the lung, the difference in weight was transient and disappeared until day 11. Following intraperitoneal infection, we also observed more pronounced yet transient weight loss in *Acod1* ^−/−^ mice (Fig 4B).

The phenotype of *Myd88* ^−/−^ mice was characterized by a high NMII burden and attenuated weight loss (Kohl *et al*, 2019), which illustrates that a higher *C. burnetii* load does not inevitably lead to an aggravation of clinical symptoms. Instead, the degree of weight loss may correlate with the production of inflammatory cytokines and immune mediators. Indeed, infected *Acod1* ^−/−^ mice expressed higher mRNA levels of *Il6*, *IFNγ*, *Nos2* and *Gbp1* than wild‐type controls in the lungs, but also in the spleen, after intratracheal infection (Fig 4C and D). A similar pattern of accentuated expression of *IFNγ* and *Nos2* was observed after intraperitoneal infection in spleen and liver, whereas *Gbp1* was not different between genotypes on day 7 (Fig 4E). On day 11 after intraperitoneal infection, levels of *Il6*, *IFNγ*, *Nos2* and *Gbp1* were higher in the liver, but not in the spleen, of *Acod1* ^−/−^ mice (Fig 4F). At the protein level, higher IFNγ was confirmed in the livers of *Acod1* ^−/−^ mice on day 11 after i.p. infection (Fig 4G), which correlated with the bacterial burden at this time (Fig 3F). However, since NMII load was comparable in the spleen and livers of *Acod1* ^−/−^ and wild‐type mice after intratracheal infection (Fig 3A and B), these data indicate that ACOD1 exerts an immunoregulatory function as described previously in mice following endotoxin challenge or infection with *M. tuberculosis* (Mills *et al*, 2018; Nair *et al*, 2018). The enhanced production of IFNγ and of several of its anti‐microbial target genes between day 7 and 11 after infection may explain the ability of *Acod1* ^−/−^ mice to resolve NMII infections despite an initial increase in bacterial replication.

### Exogenous itaconate restores intracellular ITA levels in ACOD1‐deficient macrophages and restricts *C. burnetii* replication

**Figure 5:**
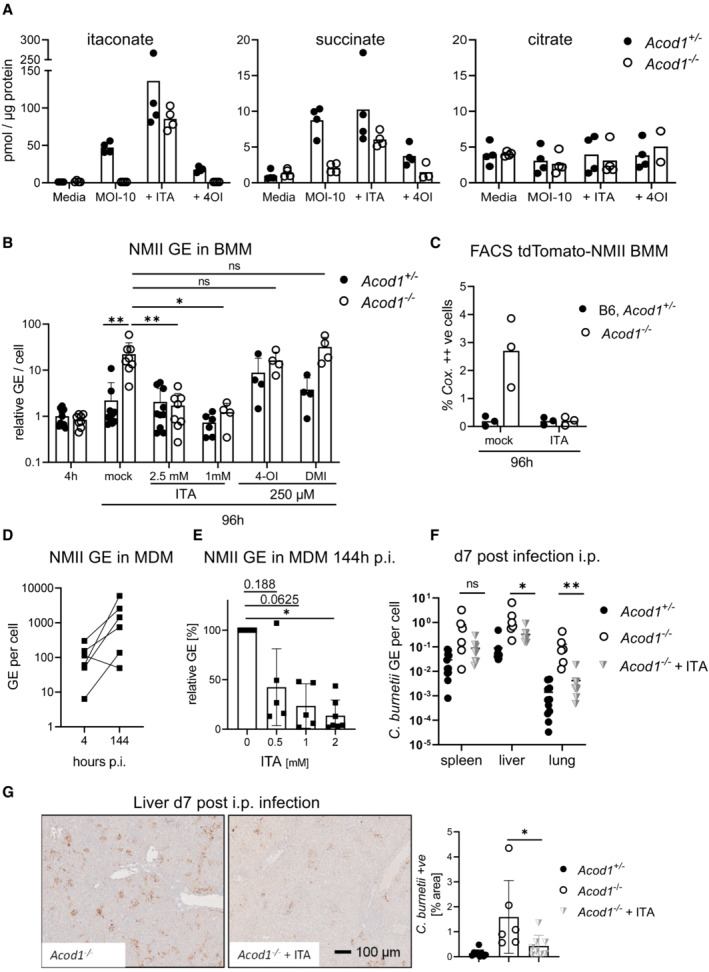
Exogenous ITA restores intracellular levels in ACOD1‐deficient macrophages and restricts *Coxiella burnetii* replication AGC–MS analysis of TCA cycle metabolites and ITA in BMM infected with NMII (MOI 10). BMM were infected for 4 h, followed by a washing step. Where indicated, exogenous ITA was added at 2.5 mM ITA, 4‐OI at 250 μM, after removing extracellular bacteria. Macrophages were harvested after 24 h. Data shown are from two independent experiments (*n* = 4 biological replicates).BITA, but not 4‐OI or DMI, reduce NMII replication to WT levels in ACOD1‐deficient BMM. Shown are qPCR data as *C. burnetii* GE normalized per cell (each dot represents one mouse and data were pooled together from 4 individual experiments with 2.5 mM ITA; *n* = 8–9 mice per genotype) and two experiments with 1 mM ITA, 250 μM 4‐OI and 250 μM DMI (*n* = 4 mice per genotype). Each dot represents one mouse. Bars show mean plus SD. *P < 0.05, **P < 0.01 in Kruskal–Wallis test with Dunn's multiple comparisons test.CBMM were infected with tdTomato‐expressing NMII and analyzed by flow cytometry 96 h after infection. ITA was added at 2.5 mM. Depicted is the percentage of BMM highly positive for tdTomato (as in Fig 2D). Pooled data from two independent experiments, each dot represents mean values of duplicates from BMM of one mouse.DNMII replication in human MDM. Infection was performed with MOI 10 for 4 h followed by washing. Cells were lysed then directly or after 144 h. Average values of replicate wells from 6 individual donors.EAddition of ITA inhibits NMII replication in human macrophages. ITA at the indicated concentrations was added to MDM after infection with NMII MOI 10. Bacterial burden was determined after 144 h as GE per cell by qPCR. Data were normalized to MDM infected but not treated with ITA (100%). Average values of replicates from *n* = 5–7 donors, error bars indicate SD. *P*‐values (Wilcoxon matched‐pairs signed rank test) are indicated.F, GTreatment of mice with 1 mg of ITA i.p. on days 1, 3, and 5 after i.p. infection with NMII lowers bacterial burden in the organs on day 7. *C. burnetii* GE quantified by qPCR (F), representative images of liver sections and quantification of immunohistochemistry for *C. burnetii* in liver sections as in Fig 3E (G). *n* = 6–11 mice (for NMII infections), pooled from two experiments. Each dot represents one mouse. Bars show mean with SD. Asterisks indicate significance (Mann–Whitney test comparing treatment and control group of NMII infected *Acod1* ^−/−^ mice in specified organs, *P < 0.05, **P < 0.01).

**Figure EV2:**
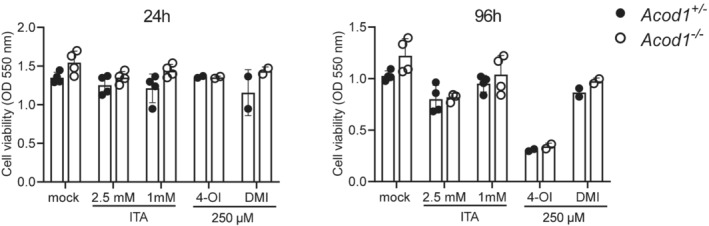
MTT Assay of BMM treated with ITA, 4‐OI and DMI MTT assay performed 24 and 96 h after addition of ITA, DMI and 4‐OI to BMM infected with NMII. Each dot represents one mouse and data were pooled together from two individual experiments with ITA (*n* = 4 mice per genotype) and one experiment with 250 μM 4‐OI and 250 μM DMI (*n* = 2 mice per genotype). Bars show mean and SD.

To investigate the impact of *Acod1* deletion on macrophage ITA production and TCA cycle metabolites, we performed gas chromatography–mass spectrometry (GC–MS)‐based analysis of mouse BMM infected with NMII (Fig 5A). While ITA was hardly detectable (<4 pmol/μg protein) in resting macrophages, NMII infection strongly increased the intracellular ITA levels (ca. 50 pmol/μg protein) in *Acod1* ^+/−^ but not in *Acod1* ^−/−^ macrophages. Consistent with the known inhibition of succinate dehydrogenase (SDH) by ITA (Cordes *et al*, 2016; Murphy & O'Neill, 2018), accumulation of succinate was observed in infected macrophages in an ACOD1‐dependent manner. To test the assumption that increased replication of NMII in *Acod1* ^−/−^ macrophages is directly caused by a lack of ITA, we performed complementation experiments by adding exogenous ITA. Due to its polar nature, the capacity of ITA to pass the cell membrane and reach significant intracellular levels in macrophages has been questioned (Mills *et al*, 2018). We employed ITA itself and, in addition, its derivatives 4‐octyl‐itaconate (4‐OI) and dimethyl‐itaconate (DMI), which were previously used by others to achieve intracellular delivery (Mills *et al*, 2018; Zhang *et al*, 2021). Remarkably, ITA added at a concentration of 2.5 mM achieved high intracellular levels in both *Acod1* ^+/−^ and *Acod1* ^−/−^ macrophages, which in fact exceeded endogenous levels (Fig 5A, left panel). In contrast, neither addition of 4‐OI (Fig 5A) nor of DMI (both used at 250 μM) led to detectable ITA peaks, indicating that they were not converted to ITA intracellularly. These initially unexpected results on the uptake or intracellular delivery of ITA in macrophages are consistent with recent data from the Artyomov group that also did not find conversion of 4‐OI or DMI to ITA in macrophages (Swain *et al*, 2020). Exogenous ITA, but not 4‐OI or DMI, increased succinate also in *Acod1* ^−/−^ BMM (Fig 5A). Intracellular levels of citrate were not altered by *Acod1* genotype or addition of ITA (Fig 5A). Exogenous ITA efficiently enabled *Acod1* ^−/−^ macrophages to restrict replication of *C. burnetii* at concentrations of 1 and 2.5 mM (Fig 5B), which showed no or little toxicity on macrophages based on MTT conversion assays (Fig EV2). In contrast, 4‐OI and DMI were highly toxic and did not significantly reduce NMII GE in *Acod1* ^−/−^ macrophages, respectively (Figs 5B and EV2). Treatment of *Acod1* ^−/−^ macrophages infected by the tdTomato‐expressing NMII strain with ITA prevented the formation of highly fluorescent cells containing large CCVs (Fig 5C). Together, GC‐MS analysis and addition of exogenous ITA demonstrated that uncontrolled replication of NMII in the absence of ACOD1 was directly due to a lack of intracellular ITA and not caused by other possible consequences of ACOD1‐deletion such as the accumulation of citrate.

**Figure EV3:**
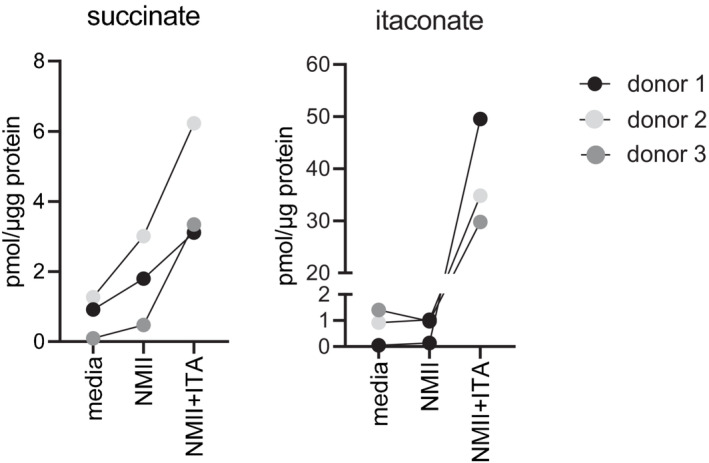
GC‐MS analysis of itaconate and succinate in human macrophages GC–MS analysis of itaconate and succinate levels in human MDM 24 h after infection. MDM were infected with NMII (MOI 10) for 4 h, followed by a washing step. Where indicated, ITA was added (2 mM) after removal of extracellular bacteria. Macrophages were harvested 24 h after infection. Data shown are from three independent stimulation using different donors.

We next asked whether the ACOD1‐ITA pathway is also relevant for the control of NMII in human macrophages. A 10‐fold increase in the number of intracellular bacteria was observed 144 h after infection of human monocyte‐derived macrophages (MDM) with NMII (Fig 5D). Despite robust induction of ACOD1 mRNA in primary MDM infected with NMII (Fig 1C), no significant endogenous itaconate could be detected by GC–MS, while treatment of MDM with exogenous ITA led to high intracellular levels (Fig EV3). Replication of NMII in MDM was blocked dose‐dependently by the addition of 0.5 to 2 mM ITA (Fig 5E), similar to the effect of ITA in *Acod1* ^−/−^ mouse BMM (Fig 5B). Succinate increased in all three donors after addition of ITA, consistent with the known inhibition of SDH; interestingly, succinate was also higher in MDM infected with NMII (Fig EV3), which may indicate endogenous itaconate levels below the sensitivity of our GC–MS analysis.

Given the strong direct activity of ITA against NMII, its effectiveness to inhibit intracellular replication in macrophages *in vitro*, and its low toxicity *in vivo* (Cordes *et al*, 2020), we next asked whether treatment of mice with ITA can reduce *C. burnetii* burden *in vivo*. Treatment of *Acod1* ^−/−^ mice infected with NMII with 1 mg ITA injected i.p. every other day significantly reduced the bacterial burden in liver and lung as determined by qPCR for *C. burnetii* genome equivalents (Fig 5F) or by immunohistochemistry (Fig 5G).

### Itaconate inhibits *C. burnetii* replication in axenic culture

**Figure 6:**
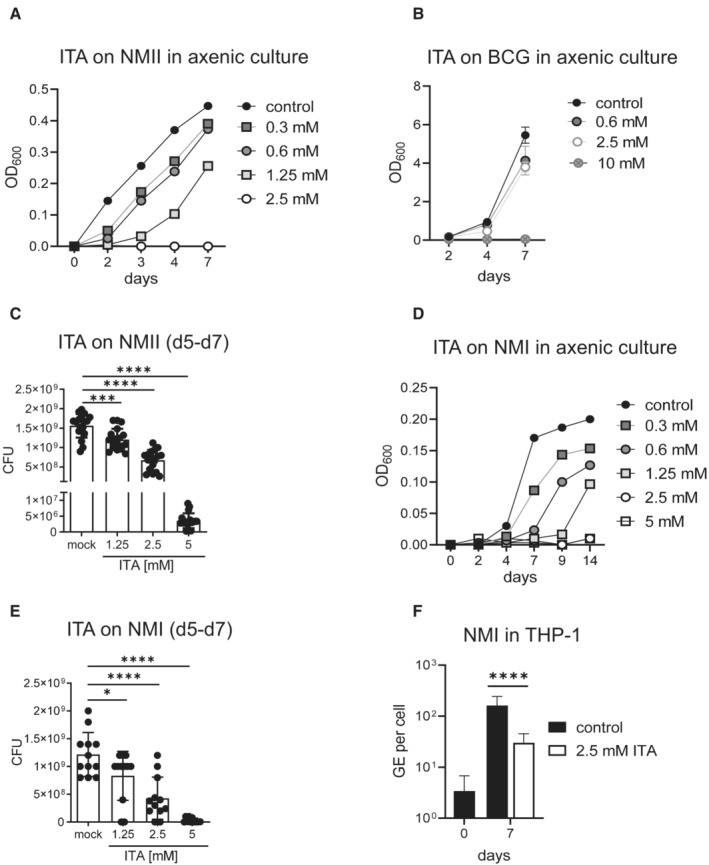
Inhibition of *Coxiella burnetii* NMII and NMI replication in axenic culture AITA inhibits NMII replication in axenic culture in ACCM‐2. Dose response and kinetic analysis. Representative of two experiments.BEffect of ITA on Mycobacterium bovis BCG. Inoculum density on d0: OD600 = 0.045. Mean and SD, *n* = 3 replicate cultures, of one representative experiment of three performed.CITA inhibits NMII growth in axenic culture after delayed addition of ITA at the indicated concentrations on day 5. On day 7, CFU were determined. Data points show results pooled from two independent experiments with three biological and technical replicates each. Bars show mean with SD. One‐way ANOVA with Dunnett's multiple comparisons test was performed. ***P < 0.001, ****P < 0.0001.D, EITA inhibits NMI replication in axenic culture. (D) Titrated ITA concentrations were added at the start of the culture in ACCM‐2. Bacterial growth was measured as OD_600nm_ at the indicated time points. Mean of triplicate cultures. (E) Effect of delayed addition of ITA on day 5 of NMI cultures on CFU determined on day 7. Data from one experiment with three biological and four technical replicates each. Bars show mean with SD. One‐way ANOVA with Dunnett's multiple comparisons test was performed. *P < 0.05, ****P < 0.0001.FTHP‐1 cells were infected with NMI (MOI 100) for 4 h, followed by washing and addition of ITA. Cells were harvested after 7 days of culture. qPCR for human albumin and *C. burnetii* was performed to calculate GE per cell. Mean and SD of 8–18 biological replicates pooled from two independent experiments. ****P < 0.0001 in Mann–Whitney test.

ITA may interfere with the replication of intracellular bacteria through effects on host cell metabolism, such as the inhibition of the mitochondrial complex II in the case of *F. tularensis* (Jessop *et al*, 2020); ITA can also act directly on bacteria, as demonstrated for *M. tuberculosis* by inhibition of the glyoxylate shunt enzyme isocitrate lyase (Michelucci *et al*, 2013). Therefore, we determined whether ITA impaired directly the replication of NMII. Addition of ITA to axenic cultures of NMII showed a dose‐dependent inhibition of bacterial growth at concentrations between 0.3 and 2.5 mM (Fig 6A), which are comparable to those found to inhibit replication in infected macrophages (Fig 5B). NMII was more sensitive to inhibition by ITA than *M. bovis* BCG whose growth was inhibited only at concentrations above 2.5 mM (Fig 6B). Of note, ITA did not only prevent replication of NMII when added at the beginning of the culture, but it also reduced bacterial numbers when used to treat established cultures between day 5 and day 7 (Fig 6C). Importantly, while ITA at 1.25 and 2.5 mM inhibited further bacterial replication, the higher concentration of 5 mM ITA reduced the CFUs by a factor of more than 300, indicating bactericidal activity (Fig 6C).

**Figure EV4:**
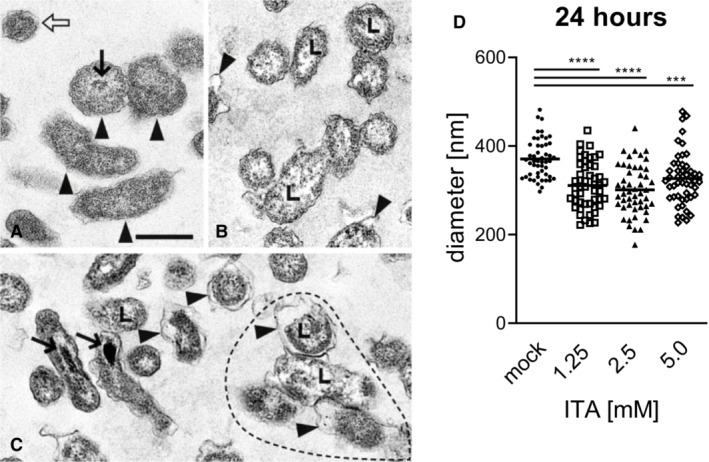
Ultrastructure of *Coxiella burnetii* NMII from axenic cultures after 24 h A–C(A) without ITA: Normal morphology of four LCV (arrowheads) and one SCV (open arrow). Note centrally located nucleoid (thin arrow). (B) with 1.25 mM ITA: LCV with unevenly distributed cytoplasm, clumped at the cytoplasmic membrane and lytic centers (L, examples). Some LCV have dilated periplasmic spaces (arrowheads). (C) with 5.0 mM ITA: LCV have markedly reduced diameters compared with controls in (A) and dilated periplasmic spaces (arrowheads). Their cytoplasm is clumped and centers are lytic as in (B) (L, examples). Four distorted LCV form an aggregate (surrounded by hatched line). Two LCV have electron dense calcium deposits in the area of the nucleoid (arrows). (A), (B), (C) are of the same magnification, scale bar = 500 nm.DQuantification of LCV diameters after treatment of *C. burnetii* NMII with ITA. Images from electron microscopy were analyzed by random selection of 50 bacterial LCV for measurement of the cell diameter. Each dot represents one cell, mean values are indicated. Statistical analysis was done by One‐way ANOVA, with comparison to the mock condition, followed by Dunnett's correction for multiple testing. Asterisks indicate *P* = 0.0001 (***) and *P* < 0.0001 (****).

To obtain insight into the effect of ITA on the ultrastructural morphology of *C. burnetii*, transmission electron‐microscopy was performed in axenic cultures of NMII after treatment with ITA. Both large (LCV) and small cell variants (SCV) of *C. burnetii* were present in the control (Fig EV4A). LCV had evenly distributed, moderately electron dense cytoplasm. Delicate electron dense nucleoid structures were present in the center surrounded by a more electron lucent halo. The outer cell membrane was close‐fitting the plasma membrane in most LCV. SCV had a homogeneous, more electron dense cytoplasm than LCV and often a pleated outer cell membrane.

**Figure EV5:**
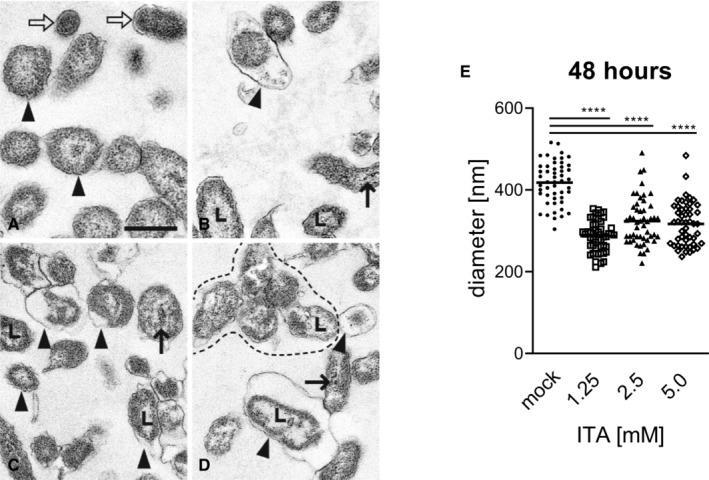
Ultrastructure of *Coxiella burnetii* NMII from axenic cultures after 48 h A–D(A) without ITA: Normal morphology of several LCV (arrowheads, examples) and two SCV (open arrows). (B) with 1.25 mM ITA: LCV with unevenly distributed cytoplasm, clumped at the cytoplasmic membrane and lytic centers (L). One LCV is markedly shrunken and has a severely dilated periplasmic space (arrowhead). Electron dense calcium deposits in the area of a nucleoid (thin arrow). (C) with 2.5 mM ITA: Many shrunken LCV with dilated periplasmic spaces (arrowheads). Some have lytic centers (L), others electron dense calcium deposits in the area of the nucleoid (thin arrow). (D) with 5.0 mM ITA: Shrunken LCV with dilated periplasmic spaces (arrowheads). Some have lytic centers (L), others electron dense calcium deposits in the area of the nucleoid (thin arrow). Distorted LCV form an aggregate (surrounded by hatched line). (A), (B), (C), (D) are of the same magnification, scale bar = 500 nm.EQuantification of LCV diameters after treatment of *C. burnetii* NMII with ITA. Images from electron microscopy were analyzed by random selection of 50 bacterial LCV for measurement of the cell diameter. Each dot represents one cell, mean values are indicated. Statistical analysis was done by One‐way ANOVA, with comparison to the mock condition, followed by Dunnett's correction for multiple testing. Asterisks indicate *P* < 0.0001 (****).

Morphologic changes were already seen 24 h after addition of ITA in the metabolically active LCV. They were comparable at concentrations of 1.25 mM (Fig EV4B) and 2.5 mM ITA (not depicted) and more severe at 5 mM ITA (Fig EV4C). In affected LCV, a variable degree of overall shrinkage of the cell body was observed (Fig EV4D), causing dilation of the periplasmic space between the cytoplasmic membrane and the outer cell membrane. The cytoplasm was unevenly distributed, clumped along the cytoplasmic membrane and more electron dense. The center was lytic without nucleoid structures. At a concentration of 5 mM ITA, some LCV had highly electron dense material located in the centers which was interpreted as deposits of calcium apatite crystals. Calcium deposits are a frequent finding in degenerate and necrotic cells (Cheville, 2009), and suggest death of the NMII cells affected. At 48 h (Fig EV5A–E), many cells in the preparation had the diameter of SCV, but comparable lesions like LCV at 24 h and were interpreted as severely shrunken LCV. They were often pleomorphic and formed aggregates (Figs EV4C and EV5D). Overall, ultramorphological changes increased with ITA concentration and time, they are compatible with degeneration of *C. burnetii*, including a bactericidal effect indicated by the intracellular calcifications.

Finally, we addressed whether ITA also is active against the virulent strain *C. burnetii* Nine Mile Phase I RSA 439 (NMI). Addition of ITA at titrated concentrations to NMI to axenic cultures dose‐dependently inhibited growth when added at the start of the culture (Fig 6D) and efficiently reduced the number of CFU when added to established cultures on day 5 (Fig 6E). These results showed a very similar activity of ITA on virulent NMI as observed before for the attenuated NMII (Fig 6A and C). The impact of exogenous ITA on intracellular replication of virulent *C. burnetii* NMI in macrophages was studied using human PMA‐differentiated THP‐1 macrophages. Replication of NMI measured after 7 days of culture was significantly and robustly inhibited by 2.5 mM ITA (Fig 6F), very similar to the effect observed for 2 mM ITA in primary human MDM infected with the NMII strain (Fig 5E).

## Discussion

This study shows that *C. burnetii* infection strongly induces *Acod1* expression which turned out to be essential for the control of NMII replication in macrophages *in vitro* and a key control mechanism in the early phase of infection *in vivo*. Notably, the effect of deletion of *Acod1* on *C. burnetii* replication was much stronger than the impact of deficiencies of several other IFNγ‐induced antimicrobial defense mechanisms (NOS2, IDO1/2, GBPs), thus establishing ACOD1 as a major protective determinant in the host defense against Q fever.

It is well known that ACOD1 catalyzes the generation of ITA by decarboxylation of cis‐aconitate. Our results from GC‐MS analyses showed that *Coxiella*‐induced *Acod1* expression in macrophages led to high‐level ITA production, which was absent in *Acod1*‐deficient cells. Thus, ACOD1‐mediated ITA generation appears to be required for curbing *C. burnetii* growth in macrophages. Since the first report of inhibition of *M. tuberculosis* by ITA (Michelucci *et al*, 2013), a similar effect has been shown for other intracellular bacteria, including *M. avium* (Gidon *et al*, 2021), *L. pneumophila* (Naujoks *et al*, 2016), *Salmonella (S.) typhimurium* (Chen *et al*, 2020), *Brucella melitensis* (Demars *et al*, 2021; Lacey *et al*, 2021) and *F. tularensis* (Jessop *et al*, 2020), but also for the extracellular bacteria *Escherichia (E.) coli* (Duncan *et al*, 2021) and *Staphylococcus (S.) aureus* (Singh *et al*, 2021). Remarkably, the ITA concentrations that we found to be required for inhibition of *C. burnetii* growth in macrophages or axenic cultures (0.6–2.5 mM), were lower than those documented for the above‐mentioned pathogens (>5 mM).

The mechanism of action for the antibacterial activity of ITA is incompletely understood and appears to be diverse. Firstly, ITA can act indirectly by altering cellular metabolism of the host cell and promote antimicrobial effector functions, such as mitochondrial ROS production (Hall *et al*, 2013). In the case of *F. tularensis*, even high concentrations of ITA (50 mM) were ineffective in axenic culture, but inhibition of mitochondrial complex II (which is identical to SDH) by 4‐OI and dimethyl‐malonate in macrophages was associated with inhibition of *F. tularensis* replication (Jessop *et al*, 2020). We have confirmed that ACOD1‐mediated ITA production leads to high intracellular succinate levels in macrophages infected with *C. burnetii*, but have not addressed whether ROS production was affected in our system.

Secondly, ITA can be directly inhibitory to bacterial replication through different mechanisms. The enzyme isocitrate lyase (ICL) is required for the glyoxylate pathway and is a target for ITA in *M. tuberculosis* (Michelucci *et al*, 2013) and *B. melitensis* (Demars *et al*, 2021). In addition, the ITA metabolite itaconyl‐CoA inhibits methylmalonyl‐CoA mutase of *M. tuberculosis* to interfere with propionate‐dependent growth (Ruetz *et al*, 2019). For other bacteria, the mechanism of growth inhibition by ITA is unknown to date. Given the acidic conditions in the phagolysosome, the recent demonstration of synergy between ITA and low pH for blocking replication of *E. coli* and *S. typhimurium* (Duncan *et al*, 2021) is of interest.

Several lines of evidence in our results indicate that ITA generated in infected macrophages directly inhibits the replication of *C. burnetii*. Firstly, exogenous ITA provided to *Acod1* ^−/−^ macrophages at non‐toxic concentrations complemented intracellular ITA levels and inhibited NMII growth within macrophages. Secondly, deficiency in *Acod1* and exogenous ITA did not alter the intracellular levels of citrate, making indirect effects through accumulation or depletion of this essential substrate for *C. burnetii* growth unlikely. Thirdly, ITA impaired the growth of NMII in axenic culture media at concentrations between 0.6 and 2.5 mM, which are much lower than for the ITA‐controlled pathogens mentioned above and are easily achieved intracellularly in activated and bacteria‐infected murine macrophages (Chen *et al*, 2020).

As ACOD1 is localized in mitochondria, newly generated ITA needs to reach the CCVs to exert its effect. In *Salmonella*‐infected macrophages, this transport is facilitated by the GTPase Rab32 that interacts with ACOD1 and is required for delivery of ITA to the *Salmonella*‐containing vacuole (Chen *et al*, 2020). It is likely that positioning of ACOD1 in the vicinity of the pathogen‐containing vacuole results in even higher local concentrations so that a direct bactericidal effect is conceivable as observed here for *C. burnetii* in axenic culture with 5 mM ITA. *C. burnetii* does not utilize the glyoxylate shunt and lacks the enzyme ICL (Seshadri *et al*, 2003). To obtain insight into the effects exerted by ITA in *C. burnetii*, we performed analysis by transmission electron microscopy which revealed changes in the ultramorphology of NMII treated with ITA that are compatible with degeneration (shrinkage of cells, clumped cytoplasm and lytic centers) and cell death (calcification). However, the exact molecular mechanism of the inhibitory effect of ITA on *C. burnetii* replication and metabolism remains to be elucidated in future work.

Importantly, we validated inhibition of *C. burnetii* replication by ITA using the virulent NMI strain, both in axenic culture and in infected human THP‐1 macrophages. The similar activity of itaconate on replication of NMI and NMII strains indicates a mechanism of action that does not involve the specific lipopolysaccharide structures in the bacterial cell wall that differ between virulent NMI and attenuated NMII (Beare *et al*, 2018). A limitation of our study is that *in vivo* infections could only be performed using the NMII strain in our BSL‐2 level animal facility. It will therefore be important to investigate the role of the ACOD1‐itaconate pathway in infection with virulent *C. burnetii* NMI in future work. However, based on the comparable activity of ITA on NMI and NMII replication in cell‐free culture and in macrophages *in vitro*, it is reasonable to expect that the ACOD1‐itaconate pathway will play a similar role during infection with virulent *C. burnetii* NMI *in vivo*.

The immunoregulatory activity of ACOD1‐ITA in sepsis and inflammation models has received more attention than its antimicrobial effects (Mills *et al*, 2018; Nair *et al*, 2018; Swain *et al*, 2020). *Acod1* ^−/−^ mice infected with NMII showed significant weight loss and increased inflammatory gene expression compared with B6 or *Acod1* ^+/−^ controls. These differences are difficult to interpret, as they may be caused by the increased bacterial load. However, two observations indicate that ACOD1 not only shows direct antimicrobial activity on *C. burnetii* but indeed has immunoregulatory effects in Q fever: firstly, we previously found in *Myd88* ^−/−^ mice that increased bacterial burden does not *per se* lead to stronger inflammatory responses; and secondly, the NMII burden in the spleen after intratracheal infection was not increased in *Acod1* ^−/−^ mice, but expression of *Il6*, *IFNγ* and *Gbp1* was still significantly higher (Fig 4D). Thus, although we interpret the unimpeded growth of NMII during the first week of infection as the major driver of inflammation and clinical disease in *Acod1* ^−/−^ mice, the loss of immunoregulatory functions of ACOD1 may contribute.

The increased *C. burnetii* burden and weight loss after intratracheal infection was transient and control of infection after day 7 coincided with the expected generation of adaptive immunity. The strong expression of IFNγ‐induced antimicrobial programs, as indicated by the robust upregulation of gene expression *in vivo* of *IFNγ* itself on day 7, of IFNγ protein in liver tissue on day 11, and of *Nos2* and *Gbp1* mRNA on day 11 after infection (by both intratracheal and intraperitoneal route), likely compensates for the lack of ITA in *Acod1* ^−/−^ mice at this later phase of infection. This notion is supported by our observation that stimulation of *Acod1* ^−/−^ BMM with IFNγ enables them to control the growth of *C. burnetii* (Fig 2D).

Treatment with ITA in mice was recently shown to improve the outcome in a reperfusion injury model (Cordes *et al*, 2020) and to counteract lung fibrosis when inhaled (Ogger *et al*, 2020). In addition, administration of DMI protected from brain injury in cerebral ischemia (Kuo *et al*, 2021). Further, application of 4‐OI in a murine endophthalmitis model caused by *S. aureus* infection synergized with antibiotics to reduce bacterial burden and attenuated inflammatory damage (Singh *et al*, 2021). The successful treatment of *Acod1* ^−/−^ mice with intraperitoneal ITA injections shown here demonstrates that sufficiently high concentrations can be achieved *in vivo* to inhibit the growth of *C. burnetii*. While in wild‐type mice, the high levels of endogenously produced ITA may not be further increased by ITA treatment, the situation is probably different in humans. Based on published results (Michelucci *et al*, 2013) and our data (Fig EV3), human macrophages produce much less ITA than their murine counterparts. This species‐difference in ITA generation might explain why exogenous ITA in our experiments was effective in reducing bacterial load of NMII in human MDM (Fig 5F) and of NMI in THP‐1 (Fig 6F) macrophages, whereas no inhibition was observed in *Acod1*‐expressing murine macrophages (Fig 5B). If this low‐level of ITA in humans compared with mice also occurs *in vivo* during infection, it would result in a state of functional, relative itaconate deficiency. In this situation, treatment with exogenous ITA would be expected to boost the capacity of infected macrophages to control *C. burnetii*. Thus, our results suggest that ITA should be further explored as a therapeutic strategy in acute and chronic Q fever.

## Materials and Methods

### Reagents

Itaconate (ITA) and its derivatives 4‐octyl itaconate (4‐OI) and Dimethyl itaconate (DMI) were purchased from Sigma‐Aldrich (Deisenhofen,Germany; ITA, I29204‐100G; 4‐OI, SML2338‐25 mg; DMI, 592498‐25G) as powder. Stock solutions were prepared in PBS (ITA and DMI) or DMSO (4‐OI).

### Mice

All mice utilized in this study were at least 6 weeks of age. C57BL/6 wild‐type mice were obtained from Charles River Breeding Laboratories (Sulzfeld, Germany) or bred at the Präklinische Experimentelle Tierzentrum of the University Hospital Erlangen (PETZ). *Acod1* ^−/−^ and *Nos2* ^−/−^ mice were from Jackson Laboratory (C57BL/6NJ‐*Acod1* ^*em1(IMPC)J*^/J and B6.129P2‐*Nos2* ^*tm1Lau*^/J). *Tnf* ^−/−^ mice (Korner *et al*, 1997) were obtained from Dr. H. Körner (University of Tasmania, Australia), *Ifnar1* ^−/−^ mice (Muller *et al*, 1994) from Dr. U. Kalinke (TWINCORE, Hannover, Germany). *Myd88* ^−/−^ mice (*Myd88* ^*tm1Ak*i^) were provided by Dr. S. Akira (University of Osaka, Japan). *Gbp2* ^−/−^ mice (*Gbp2* ^*tm1.1Kpf*^) were provided by Dr. K. Pfeffer (Universitätsklinikum Düsseldorf, Germany) (Degrandi *et al*, 2013). Gbp(chr3) mice lacking *Gbp1*, *Gbp2*, *Gbp3*, *Gbp5* and *Gbp7* (Del(3Gbp2‐ps‐Gbp5)1Ktak) (Yamamoto *et al*, 2012) were obtained from Dr. Thomas Henry (University of Lyon, France). Mice with a combined deletion in *Ido1* and *Ido2* (*Ido1‐Ido2* ^*ΔΔ*^) (Van de Velde *et al*, 2016) were generated and provided by one of us (P.M.). All mice were generated on a C57BL/6 background or crossed back multiple generations to C57BL/6.

### Culture of *Coxiella burnetii*

#### *Coxiella burnetii* Nine Mile phase II (NMII)

An isolate of the *C. burnetii* Nine Mile phase II (NMII) strain clone 4 (NMII, RSA493) was generously provided by Matteo Bonazzi (Institut de Recherche en Infectiologie de Montpellier, Montpellier, France). One aliquot of purified NMII was propagated in a 75 cm^2^ tissue culture flask containing 30 ml acidified citrate cysteine medium (ACCM‐2, 4,700–003, Sunrise Science Products, San Diego, CA, USA) at 37°C in a humidified atmosphere of 5% CO_2_ and 2.5% O_2_ (Omsland *et al*, 2009). After 4 days of culture, NMII were transferred overnight to room temperature and ambient atmosphere. Subsequently, bacteria were pelleted for 15 min at 4,500 *g*, resuspended in 1 ml phosphate buffered saline (PBS) and quantified by optical density at OD_600_, where an OD_600_ of 1 equals 1 × 10^9^ NMII per ml. Bacteria were diluted in PBS or cell culture media without antibiotics and kept on ice until use for infection of mice or macrophages.

#### *Coxiella burnetii* Nine Mile phase I RSA 439 (NMI)

It was routinely propagated in L‐929 mouse fibroblasts at 37°C under 5% CO2 in DMEM containing 20 mM L‐glutamine (Lonza, Cologne, Germany) and 5% heat inactivated fetal bovine serum (Capricon Scientific, Ebsdorfergrund, Germany) and purified as previously described (Samuel & Hendrix, 2009). Cell culture propagated bacteria were passaged once in acidified citrate cysteine medium (ACCM‐2) as mentioned elsewhere (Omsland *et al*, 2009). Briefly, ACCM‐2 was inoculated with 10^5^ genome equivalents per milliliter (GE/ml) and incubated for 7 days at 37°C with 5% CO_2_ and 2.5% O_2_. Bacteria were harvested (10,000 *g*, 4°C, 20 min) and resuspended in sucrose glycerol (270 mM sucrose, 10% (v/v) glycerol (both from Carl Roth, Karlsruhe, Germany)) for storage at −80°C. All experiments with *C. burnetii* NMI were carried out under biosafety laboratory 3 conditions.

### Generation of tdTomato‐expressing *C. burnetii*

The tandem‐di‐Tomato (tdTomato) expressing NMII strain was prepared as described previously (Matthiesen *et al*, 2020): the tdTomato gene (codons optimized for expression in *C. burnetii*) was amplified by PCR with Q5 polymerase (New England Biolabs, Frankfurt, Germany) using primers a533 (5′‐GATTTAAGAAGGAGATCTGCAGATGGTGTCAAAAGGAG‐3′) and a534 (5′‐AAGCTTGCATGCCTCAGTCGACTTATTTATAAAGTTCATCCATGC‐3′) to attach overhang‐sequences for Gibson assembly. The destination vector pJB‐CAT‐ProD‐mCherry (kindly provided by Robert Heinzen and Paul Beare, Rocky Mountain Laboratories, MT, USA) was digested with PstI and SalI. The Gibson assembly method was utilized to ligate the purified PCR product with the digested vector to create the *C. burnetii* expression vector pJB‐CAT‐ProD‐tdTomato, which was subsequently used to electroporate and transform *C. burnetii* in ACCM‐D medium as described before (Omsland *et al*, 2009; Schafer *et al*, 2017). Transformed cells were plated on ACCM‐D agar plates containing 3 μg/ml chloramphenicol (CA) and were incubated for 10–14 days. The screening for positive clones was done via PCR using primers a535 (5′‐CACAGCTAACACCACGTCGTCC‐3′) and a537 (5′‐CTGCATCACTGGCCCATCGG‐3′). Positive clones were expanded in liquid ACCM‐D in presence of 3 μg/ml CA and analyzed for fluorophore expression via fluorescence microscopy.

### Treatment of *C. burnetii* with itaconate

Five ml of ACCM‐2 media were inoculated with *C. burnetii* NMII or *C. burnetii* NMI at a concentration of 10^6^ *Coxiella*/ml and propagated at 37°C, 5% CO_2_ and 2.5% O_2_. At different time points post‐inoculation the bacteria were treated with ITA for the indicated time periods. As controls, PBS was added to the bacterial culture. For determination of colony forming units (CFU), the bacterial culture was centrifuged at 4,500 *g* for 15 min and the pellet was resuspended in 200 μl ACCM‐D. Ten μl of serial dilutions were dropped on ACCM‐D/0.3% agarose plate in triplicates. The plates were incubated for 2 weeks at 37°C, 5% CO_2_ and 2.5% O_2_. CFU were calculated according to the corresponding dilution factor. To measure bacterial growth by optical density, *C. burnetii* cultures were propagated as mentioned above. At the indicated time points, 100 μl were directly taken from the culture to determine the optical density at OD_600_.

### Mouse macrophages

Primary bone marrow‐derived macrophages (BMM) were propagated from the bone marrow of femurs and tibiae by culture in petri dishes in complete Dulbecco's modified Eagle medium (cDMEM; DMEM [Life Technologies] plus 10% fetal bovine serum [FBS; Biochrom, Berlin, Germany], 50 μM β‐mercaptoethanol, penicillin and streptomycin) complemented with 10% L929 cell‐conditioned medium [LCCM] as a source of macrophage colony‐stimulating factor (M‐CSF) at 37°C, 5% CO_2_ and 21% O_2_ for 7 d. Non‐adherent bone marrow cells were used after over‐night culture in cDMEM with 10% LCCM, counted and plated in petri dishes at a density of 5–8 × 10^6^ per 10‐cm dish. On day 3 of culture, additional 5 ml of cDMEM with 10% LCCM were added. Adherent macrophages were harvested on day 7 by treatment with Accutase (Sigma, Deisenhofen, Germany), washed and counted.

### Human monocyte‐derived macrophages

Leukocyte Reduction System chambers were obtained from the Department of Transfusion Medicine at the University Hospital Erlangen (Ethics commission protocol number 111_12B). Peripheral blood mononuclear cells (PBMC) were separated using Biocoll separation solution and centrifugation for 30 min at 510 *g* without brakes at room temperature. The interface containing PBMC was carefully removed, transferred to a fresh tube and washed once with PBS and centrifugation at 400 *g* for 5 min at room temperature. After resuspending the pellet in PBS, platelets were removed by another centrifugation step at 90 *g* for 10 min at room temperature. Next, CD14^+^ monocytes were isolated through positive selection using Miltenyi CD14 beads and LS MACS columns. Human monocyte‐derived macrophages (MDM) were differentiated from purified monocytes by culture in complete RPMI 1640 containing 10% FBS, HEPES, penicillin/streptomycin, 50 μM mercaptoethanol, and 50 U/ml recombinant human GM‐CSF (Leukine, Genzyme). On day 3 or 4 of culture, cells were fed with fresh media and GM‐CSF, and then harvested on day 7 by incubation with Accutase diluted in PBS 1:4 ratio. After washing and counting, MDM were seeded in 96‐well plates at a density of 1.5 or 2.0 × 10^5^ cells per well, and allowed to adhere and rest over‐night in complete RPMI without antibiotics.

### *In vitro* infection of macrophages with *C. burnetii* NMII

The day before infection, murine BMM and human MDM were harvested, seeded and cultured at 37°C, 5% CO2 in DMEM (for BMM) or RPMI (for MDM) complete medium containing 10% fetal calf serum (FCS) and 50 μM β‐mercaptoethanol without antibiotics. For microscopic analysis, 0.5 × 10^6^ macrophages were seeded on 10 mm coverslips in 24‐well dishes, whereas in 96‐well dishes a density of 1.5 × 10^5^ cells/well were used for qPCR of *C. burnetii* GE and mRNA expression of host genes. The cells were infected with NMII at a MOI of 10, if not indicated otherwise. Following infection, cells were incubated at 37°C and 5% CO2. After 4 h, cells were washed with cDMEM/cRPMI media without antibiotic to remove extracellular NMII and finally supplied with antibiotic free cDMEM/cRPMI. At the indicated time points, supernatants were removed and the cells lysed in PeqDirectLysis Buffer (Peqlab/VWR 30–2010) with Proteinase K (PeqLab, Germany) for DNA or TriReagent (VWR, T9424‐100ML) for RNA preparation, respectively.

### Infection of human THP‐1 macrophages with *C. burnetii* NMI

The human monocytic cell line THP‐1 was routinely propagated in RPMI 1640 (Lonza, Cologne, Germany) containing 5% fetal bovine serum at 37°C under 5% CO_2_. For differentiation into macrophages, THP‐1 cells were treated with 200 nM phorbol 12‐myristate‐13‐acetate (PMA, Sigma‐Aldrich) for 24 h. Growth assays were performed in 24 well plates with 5 × 10^5^ PMA‐treated cells/ml. A MOI of 100 was used and non‐internalized bacteria were removed after 4 h post infection by washing three times with PBS. The ability of NMI to grow in THP‐1 cells with or without the addition of 2.5 mM itaconate was monitored by qPCR directly after infection and on day 7 post infection. To this end, non‐internalized bacteria were removed by washing and the cell layer was harvested in 200 μl PBS by scraping.

### Quantification of *C. burnetii* NMI by qPCR

Bacteria were quantified by real time PCR (qPCR) targeting the isocitrate dehydrogenase encoding gene icd according to Klee *et al* (2006). Briefly, PCR reactions were carried out using Maxima Probe/ROX qPCR Master Mix (ThermoFisher Scientific, Waltham, MA, USA), 300 nM each primer (icd‐439F [5′‐CGTTATTTTACGGGTGTGCCA‐3′] and icd‐514R [5′‐CAGAATTTTCGCGGAAAATCA‐3′]), 100 nM probe (icd‐464TM [FAM‐CATATTCACCTTTTCAGGCGTTTTGACCGT‐TAMRA‐T]) and 2 μl of a 500 bp icd gene fragment as standard or sample. The qPCR was carried out on a CFX96 Touch Real‐Time Instrument (BioRad) using the following cycling conditions: 2 min at 50°C, 10 min at 95°C, followed by 45 cycles of 15 s at 95°C and 30 s at 60°C. Data collection and analysis was carried out using the CFX Maestro 2.3 software (BioRad, Munich, Germany).

### Immunofluorescence microscopy

For indirect immunofluorescence microscopy analyses, BMM were cultured on 10 mm coverslips in 24‐well dishes. At indicated points of time, cells were washed three times with equilibrated PBS, fixed for 15 min with equilibrated 4% paraformaldehyde (PFA) and permeabilized with ice‐cold methanol for 1 min. Cells were quenched and blocked with 50 mM NH_4_Cl in PBS/5% goat serum (GS) for 60 min at room temperature. Incubation with primary antibody dilution in PBS/5% GS was conducted at room temperature for 60 min. Subsequently, cells were washed three times with PBS and further incubated with secondary antibodies in PBS/5% GS for 30 min at room temperature. After final 3× washing with PBS, coverslips were mounted using ProLong Diamond containing DAPI. For visualization, a Carl Zeiss LSM 700 Laser Scan Confocal Microscope and the ZEN2009 software (Jena, Germany) were used.

In this study, we used primary antibodies directed against *C. burnetii* (polyclonal rabbit serum, Davids Biotechnologie, Regensburg, Germany) as a 1:5,000 dilution and LAMP‐1 (monoclonal rat IgG, Developmental Studies Hybridoma Bank, Iowa, IA, USA) as a 1:400 dilution. Secondary antibodies were labeled with Alexa Fluor 594 and Alexa Fluor 488, respectively (both were used as 1:800 dilution) (Dianova, Hamburg, Germany).

### Transmission electron microscopy

Bacteria suspensions were fixed 1:2 (v:v) with 2.5% glutaraldehyde in 0.1 M cacodylate buffer (pH 7.2) containing 1.8% glucose for 1 h at 4°C. The fixative was replaced by cacodylate buffer until further preparation. For embedding, bacteria were centrifuged for 15 min at 15,000 *g* in an Eppendorf centrifuge to obtain a pellet. The pellet was evenly mixed with 20 μl of 2% molten agarose on a glass slide, allowed to cool and sectioned into 1 mm^3^ cubes. Cubes were post‐fixed in 2% osmium tetroxide and embedded in Araldite Cy212. Relevant areas were selected in Toluidine‐blue‐stained semi‐thin sections. Ultrathin sections (85 nm) collected on 200 mesh copper grids were stained with uranyl acetate and lead citrate and examined by transmission electron microscopy (Tecnai 12; FEI/ThermoFisher Scientific) at 80 kV. One section per treatment group was selected to evaluate bacterial morphology. For this, representative electron micrographs were taken from six different quadrants of this section at a magnification of 6,800 x using a 4kx4k digital CMOS camera (TEMCAM FX416, TVIPS, Garching, Germany). For morphometric analysis, diameters of 50 LCV selected by chance in one 2,900× overview micrograph from each treatment group were measured using the EM‐Measure software (TVIPS, Garching, Germany).

### *In vivo* infection experiments

All mouse experiments were approved by the regional government (Regierung von Unterfranken, animal protocols 54‐2532.1‐44/13 and 55.2.2–2532.2‐854‐14). Mice were bred at the PETZ of the University Hospital Erlangen and transferred to a biosafety level 2 animal room at least 1 week before infection. Both female and male mice were used in the infection experiments. For comparison between *Acod1* ^−/−^ and B6 or *Acod1* ^+/−^ mice, groups were matched for sex and age as closely as possible. Groups of mice were infected with NMII either intraperitoneally (5 × 10^7^ CFU/200 μl PBS/mouse) or intratracheally (10^6^ CFU/50 μl PBS/mouse), as described before (Kohl *et al*, 2019). Intratracheal infection was performed by direct injection of bacterial suspension into the trachea of the mice. To do this, mice were anesthetized with isofluorane, placed in dorsal position and the trachea was accessed after incision of the skin. Prior to surgery, buprenorphine (0.1 mg/kg) was injected intraperitoneally for analgesia and mice were supplied with Butorphanol (0.12 mg/ml drinking water for 24 h after the surgery). The physical condition of the mice was monitored regularly, including measuring the weight of the animals. At the indicated time points, mice were sacrificed by cervical dislocation and organ tissue (~ 20–50 mg) of spleen, liver, and lung were collected. Tissue samples were preserved in PeqDirectLysis buffer (Peqlab, Erlangen, Germany) plus proteinase K (Roche) for DNA preparation or in RNAlater (Qiagen, Hilden, Germany) for subsequent RNA isolation. For histopathology, organ pieces were fixed in PFA 4% over night.

### DNA isolation and quantification of *C. burnetii* NMII burden via qPCR

Organ tissue or macrophages in cell culture were lysed in PeqDirectLysis buffer plus proteinase K at 56°C under shaking overnight. Then, proteinase K was inactivated at 85°C for 45 min. DNA from organ tissue or macrophages was used directly for qPCR. We defined the ratio of *C. burnetii* genomic copies to BMM genomic copies as bacterial load per cell. To amplify *C. burnetii* DNA, a TaqMan‐based quantitative PCR for the insertion sequence IS1111 was performed using 5′‐CATCGTTCCCGGCAGTT‐3′ as forward, 5′‐TAATCCCCAACAACACCTCCTTA‐3′ as reverse primer, and the internal fluorogenic probe 6FAM‐CGAATGTTGTCGAGGGACCAACCCAATAAA‐BBQ (TibMolbiol, Berlin, Germany). Host cell genomic copies were quantified from the same sample using a primer set specific for murine albumin gene (exon 7): forward, 5′‐GGCAACAGACCTGACCAAAG‐3′ and reverse, 5′‐CAGCAACCAAGGAGAGCTTG‐3′. For samples derived from human macrophages, genomic copies were determined using primers for the human albumin gene (forward, 5′‐GTGAACAGGCGACCATGCT‐3′, reverse 5′‐GCATGGAAGGTGAATGTTTCAG‐3′). Standard curves for human and murine genomes were generated by serial dilution of DNA from human PBMC or mouse spleen cells. To quantitate *C. burnetii* genome equivalents, a standard curve using titrated DNA prepared from a defined number of *in vitro* cultured NMII was included in each experiment. *C. burnetii* burden was calculated per cell by normalizing bacterial genome copy numbers to albumin copy numbers.

qPCR was carried out in 384‐well optical plates on an ABI Prism 7900HT sequence‐detection system. To quantify *C. burnetii* DNA, 2× Roche FastStart Universal Master Mix (6 μl) with 0.2 μM final concentration of each primer and an internal fluorogenic probe 20 μM was used. For the quantification of the host albumin gene we used 2× SYBRselect master mix together with 0.83 μM of each primer. For both qPCRs, 2 μl of isolated DNA (prediluted 1:4 for macrophage lysates and 1:20 for organ samples in ultrapure H_2_O) was used as template in a final volume of 12 μl per reaction.

### RNA isolation and gene expression analysis by qRT‐PCR

RNA from organ tissue (stored in RNAlater stabilizing reagent until further processing) was isolated using peqGold TriFast™ (Peqlab) or TriReagent (Sigma‐Aldrich, Deisenhofen, Germany) and cDNA was synthesized using High Capacity cDNA Reverse Transcription Kit (Applied Biosystems). Primers and probes were chosen from the Universal Probe library (Roche) for the specific gene expression as follows. *Hrpt* (mouse), forward primer 5′‐TCCTCCTCAGACCGCTTTT‐3′, reverse primer 5′‐CCTGGTTCATCATCGCTAATC‐3′, UPL probe number 95. *Il6* (mouse), forward primer 5′‐GCTACCAAACTGGATATAATCAGGA‐3′, reverse primer 5′‐CCAGGTAGCTATGGTACTCCAGAA‐3′, UPL probe number 6. *Ifnγ* (mouse), forward primer 5′‐GAACTGGCAAAAGGATGGTG‐3′, reverse primer 5′‐CTTGCTGTTGCTGAAGAAGG‐3′, UPL probe number 21. *Nos2* (mouse), forward primer 5′‐CTTTGCCACGGACGAGAC‐3′, reverse primer 5′‐TCATTGTACTCTGAGGGCTGAC‐3′, UPL probe number 13. *Acod1* (mouse), forward primer 5′‐GCTTTTGTTAATGGTGTTGCTG‐3′, reverse primer 5′‐GGCTTCCGATAGAGCTGTGA‐3′, UPL probe number 45. *Gbp1* (mouse), forward primer 5′‐AATAAGCTGGCTGGAAAGCA‐3′, reverse primer 5′‐TGTGTGAGACTGCACAGTGG‐3′, UPL probe number 7. *Gbp2* (mouse), forward primer 5′‐CCAAAAGTTCCAGACAGAATTAGG‐3′, reverse primer 5′‐ACGAGCTGATGAGACATCCA‐3′, UPL probe number 62. *Gbp4* (mouse), forward primer 5′‐GAGCAGCTCATCAAAGACCA‐3′, reverse primer 5′‐TTCCTCACGGAAAGTCTTTTG‐3′, UPL probe number 67. *Gbp5* (mouse), forward primer 5′‐CAACAGTGGATCCTGAAGCA‐3′, reverse primer 5′‐GCTGCTGTTGAAGTGCTCTG‐3′, UPL probe number 4. *Ido1* (mouse), forward primer 5′‐TTGCTACTGTTTTGAATTGTAATGTG‐3′, reverse primer 5′‐AAGCTGCCCGTTCTCAATC‐3′, UPL probe number 96. *Acod1* (human), forward primer 5′‐TTTGTGAACGGTGTGGCTAT‐3′, reverse primer 5′‐ GTGAGGACAGGAAGGACAGC‐3′, UPL probe number 87. *PPIA* (human), forward primer 5′‐CCTAAAGCATACGGGTCCTG‐3′, reverse primer 5′‐CACTTTGCCAAACACCACAT‐3′, UPL probe number 48. The fold change in gene expression was determined by the ΔΔCT method, using HPRT as housekeeping gene for calculation of ΔCT values and samples of naïve wild type or heterozygous mice as calibrators. For human samples, PPIA was used as housekeeping gene and samples were calibrated to non‐infected controls.

### Detection of IFNγ protein in tissue homogenates

Liver samples were harvested from mice and homogenized in PBS with a Star Beater (VWR) for 3 min at 30 Hz. An aliquot was removed and spun at 10,000 *g*, the supernatant was used for detection of cytokine proteins using the LEGENDplex™ Mouse Inflammation Panel (Biolegend), a multiplex assay using fluorescence‐encoded beads for analysis on flow cytometers. Processing of samples and standard curve preparation were performed according to the manufacturer's instruction. Raw data (signal intensities) were analyzed by LEGENDplex Data Analysis Software (Qognit) to obtain cytokine concentrations. To normalize for tissue size, the total protein concentration of the homogenate was determined with a BCA kit (Pierce) and pg IFNγ per mg protein was calculated.

### Processing of organ tissue for histopathological analysis

After fixation of organ pieces for 24 h in PFA 4% at 4°C tissue was washed with PBS and subsequently embedded in paraffin. In total, 5 μm paraffin‐embedded tissue slides were transferred to Star Frost microscope slides (Knittel GmbH, Braunschweig, Germany) for staining of the tissue.

### Immunohistochemistry staining of organ tissue

*C. burnetii* was detected using the α‐*C. burnetii* antiserum described above (dilution 1:2,000). Detection of bound antibodies was performed with biotinylated secondary goat anti‐rabbit antibody (dilution 1:500) and ABC‐Kit using the peroxidase substrate method DABImmPact (all from Vector Laboratories, Burlingame, CA, USA).

### Slide scanning and quantification of images

DAB‐stained tissue arrays of *C. burnetii* infected mice were digitized using a Hamamatsu digital slide scanner, model C13210, with 40× magnification, automatic exposure and focussing, resulting in a resolution of 221 nm/pixel (114932 DPI). Tissues of interest were exported at 20× magnification as TIF‐formatted tile images. Using the Color Threshold method in Image J, the area under tissue and DAB‐stained areas were recognized in the HSB color space; as tissue: Hue 0°–360°, Saturation 4–100%, Brightness 64.5–100%; DAB: Hue 280°–70°, Saturation 25–100%, Brightness 8–100%. Binary masks were created. For smoothing, the masks were enlarged three times and then reduced again three times, each time by 1 pixel per step. Outliers with a radius of 5 pixels were removed. The positively colored area of *C. burnetti* was calculated as the percentage of the DAB mask to the total tissue mask.

### GC–MS analysis

For GC‐MS analysis, BMM were seeded (1 × 10^6^ cells / well) in 6 well‐plates the day before infection in cDMEM without antibiotics. After overnight incubation, NMII was added at MOI‐10. 4 h after infection extracellular bacteria were washed away with cDMEM media, then supplied with ITA and its derivatives (4‐OI and DMI). 24 h after infection, BMM were washed 4 times with PBS (1 ml/well), with careful removal of PBS to ensure complete removal of extracellular ITA and derivatives. Macrophages were lysed with ice‐cold 80% methanol and lysates were immediately frozen at −80°C until further analysis. Sample preparation and measurement was performed as recently described (Hayek *et al*, 2019). The internal standard solution was expanded to include ^13^C_5_‐ITA (Toronto Research Chemicals/Biozol, Eching, Germany) to ensure accurate ITA quantification. Total protein amount was determined using the fluorescent dye SERVA Purple (Serva, Heidelberg, Germany) as recently described (Berger *et al*, 2021).

### Flow cytometry analysis

BMM infected with fluorescent tdTomato‐expressing *C. burnetii* were analyzed by flow cytometry at the indicated time points. After discarding the media, cells were washed with PBS/2% FCS in 24‐ or 96‐well plates according to the experimental setup. Then cells were fixed with 2% PFA for 30 min at room temperature. Following one more washing step, cells were gently scraped off the bottom of the plate in PBS/2% FBS with a pipette tip. Finally, cells were filtered through a 70 μm cell strainer into FACS tubes and analyzed using a BD LSRFortessa (BD Biosciences, San Jose, CA, USA). Single cells were gated and tdTomato fluorescence was quantified in the PE channel using FACS Diva and Flowjo software.

### MTT assay

MTT assay was performed to investigate cellular toxicity of ITA and its derivatives. At the indicated times, 20 μl of MTT (Sigma Aldrich, M2128‐5G) solution (5 mg/ml) were added to each well of a 96 well‐plate on top of the cells containing 100 μl medium. Three to five hours after incubation at 37°C, 5% CO_2_, 10% SDS in 0.01 N HCl (100 μl/well) was added to the MTT mixture and incubated overnight. Plates were measured by means of an ELISA reader at 550 nm; mean absorbance values of the wells containing media only (without cells) incubated with MTT and SDS solution were used as blank for calibration.

### Statistical analysis

Experiments were performed with biological replicates and repeated as indicated in the Figure legends. In mouse experiments, the groups were matched for age and sex as closely as possible. No blinding was done, but, when possible, automated data acquisition was used (e.g. image quantification). Samples or animals were only excluded from analysis when samples or measurements were technically failed or were missing. Statistical analysis was performed as described individually for each graph in the figure legends using GraphPad Prism (version 8). Datasets were tested for normal distribution using the D'Agostino & Pearson test. If normality was confirmed, ANOVA test was used with Dunnet's multiple comparisons; all other data were analyzed for significant differences with non‐parametric tests (Mann–Whitney, Wilcoxon, Kruskal–Wallis test with Dunn's multiple comparisons). Correction for multiple testing was also done in GraphPad Prism. Asterisks indicate different levels of significant *P* values (*P < 0.05, **P < 0.01, ***P < 0.001, ****P < 0.0001).

**The paper explained**

**Problem**

Chronic Q fever caused by the intracellular bacterium *Coxiella burnetii* is a severe infection with often lethal vascular complications. Antibiotics are frequently inefficient in chronic Q fever despite extended treatment courses. *C. burnetii* infects macrophages and replicates in the phagolysosome. The determinants of success or failure in the host immune response to *C. burnetii* are not well understood.

**Results**

In this manuscript, we identify a host‐protective role for the metabolite itaconate produced by the enzyme ACOD1 (aka IRG1) in macrophages in response to *C. burnetii*. ACOD1 mRNA was highly expressed in the spleens of mice after infection with *C. burnetii*, as well as in human and murine macrophages. Infected ACOD1‐deficient mice developed high bacterial burden and significant weight loss. Treatment with itaconate corrected this phenotype in mouse and human macrophages, and protected mice after infection *in vivo*. Mechanistically, we show a strong direct inhibitory activity of itaconate on *C. burnetii*.

**Impact**

These results establish ACOD1‐derived itaconate as an essential macrophage effector molecule for containment of *C. burnetii* and resolving infection. Given the lack of efficient antibiotic regimens for chronic Q fever patients, our findings suggest itaconate as novel host‐derived treatment strategy.

## Author contributions

**Lisa Kohl:** Conceptualization; formal analysis; investigation; writing—review and editing. **Md Nur A Alam Siddique:** Conceptualization; formal analysis; investigation; writing—review and editing. **Barbara Bodendorfert:** Formal analysis; investigation; writing—review and editing. **Raffaela Berger:** Investigation. **Annica Preikschat:** Investigation. **Christoph Daniel:** Investigation; writing—review and editing. **Martha Ölke:** Investigation. **Elisabeth Liebler‐Tenorio:** Formal analysis; investigation; writing—review and editing. **Jan Schulze‐Luehrmann:** Resources; formal analysis; investigation. **Michael Mauermeir:** Methodology. **Kai‐Ting Yang:** Investigation. **Inaya Hayek:** Resources. **Manuela Szperlinski:** Investigation. **Jennifer Andrack:** Investigation. **Ulrike Schleicher:** Resources; writing—review and editing. **Aline Bozec:** Resources. **Gerhard Krönke:** Resources. **Peter Murray:** Resources. **Stefan Wirtz:** Resources. **Masahiro Yamamoto:** Resources. **Valentin Schatz:** Resources. **Jonathan Jantsch:** Resources. **Peter, J Oefner:** Resources; writing—review and editing. **Daniel Degrandi:** Resources. **Klaus Pfeffer:** Resources. **Katja Mertens‐Scholz:** Resources; formal analysis; methodology. **Simon Rauber:** Formal analysis; investigation; writing—review and editing. **Christian Bogdan:** Writing—review and editing. **Katja Dettmer:** Formal analysis; supervision. **Anja Lührmann:** Conceptualization; supervision; methodology; writing – review and editing. **Roland Lang:** Conceptualization; formal analysis; supervision; writing – original draft; writing – review and editing.

## Disclosure and competing interests statement

The authors declare that they have no conflict of interest.

## For more information

Information about Q fever disease, epidemiology, research activities:

- https://q‐gaps.de/en/
- https://www.ecdc.europa.eu/en/q‐fever

Authors' homepage:

iii https://www.mikrobiologie.uk‐erlangen.de/forschung/forschergruppen‐arbeitsgruppen/ag‐prof‐r‐lang/

Homepage Collaborative Research Center 1,181 Checkpoints for Resolution of Inflammation:

iv https://www.sfb1181.forschung.fau.eu/

## Supporting information



Expanded View Figures PDF

PDF+
