# Supplementary material for: Macrophages inhibit Coxiella burnetii by the ACOD1‐itaconate pathway for containment of Q fever
Source: EMBO Mol Med. 2022 Dec 7;15(2):e15931. doi: 10.15252/emmm.202215931 (PMC9906395; doi:10.15252/emmm.202215931)
Supplement: Supplementary file 1 — Expanded View Figures PDF [file EMMM-15-e15931-s003.pdf]

Expanded View Figures

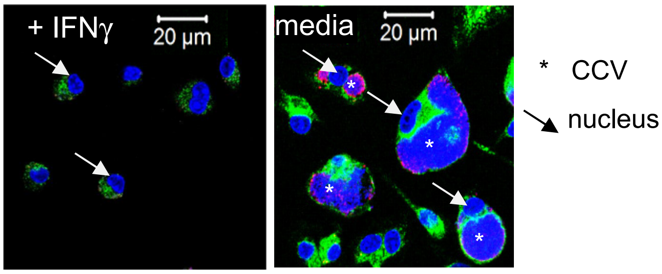

Figure EV1. Immunofluorescence microscopy of *Acod1*$^{-/-}$ BMM treated with IFNγ.

Immunofluorescence microscopy of *Acod1*$^{-/-}$ BMM 120 h after infection with NMII at MOI 10. IFNγ was added or not 4 h after infection when extracellular *Coxiella burnetii* was removed. Staining for Lamp1 appears in green, staining for *C. burnetii* in pink, DAPI stain for DNA shows nuclei (examples marked by arrows) and large CCV in *Acod1*$^{-/-}$ BMM in the absence of IFNγ (marked by asterisk).

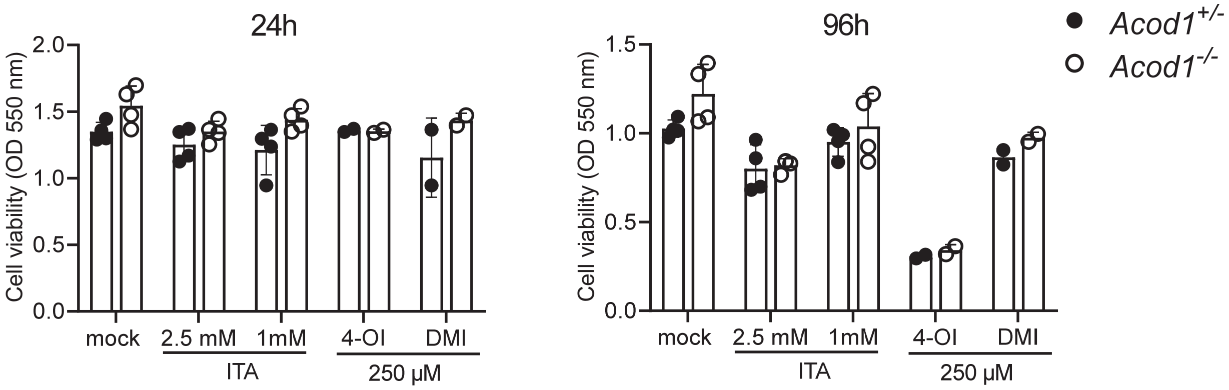

Figure EV2. MTT Assay of BMM treated with ITA, 4-OI and DMI.

MTT assay performed 24 and 96 h after addition of ITA, DMI and 4-OI to BMM infected with NMII. Each dot represents one mouse and data were pooled together from two individual experiments with ITA (n = 4 mice per genotype) and one experiment with 250 µM 4-OI and 250 µM DMI (n = 2 mice per genotype). Bars show mean and SD.

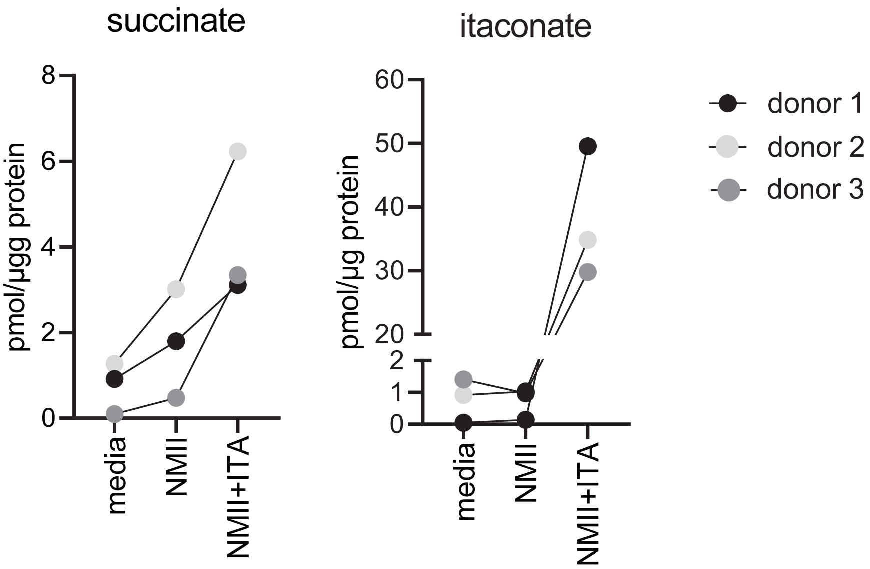

Figure EV3. GC-MS analysis of itaconate and s-uccinate in human macrophages.

GC–MS analysis of itaconate and succinate levels in human MDM 24 h after infection. MDM were infected with NMII (MOI 10) for 4 h, followed by a washing step. Where indicated, ITA was added (2 mM) after removal of extracellular bacteria. Macrophages were harvested 24 h after infection. Data shown are from three independent stimulation using different donors.

© 2022 The Authors

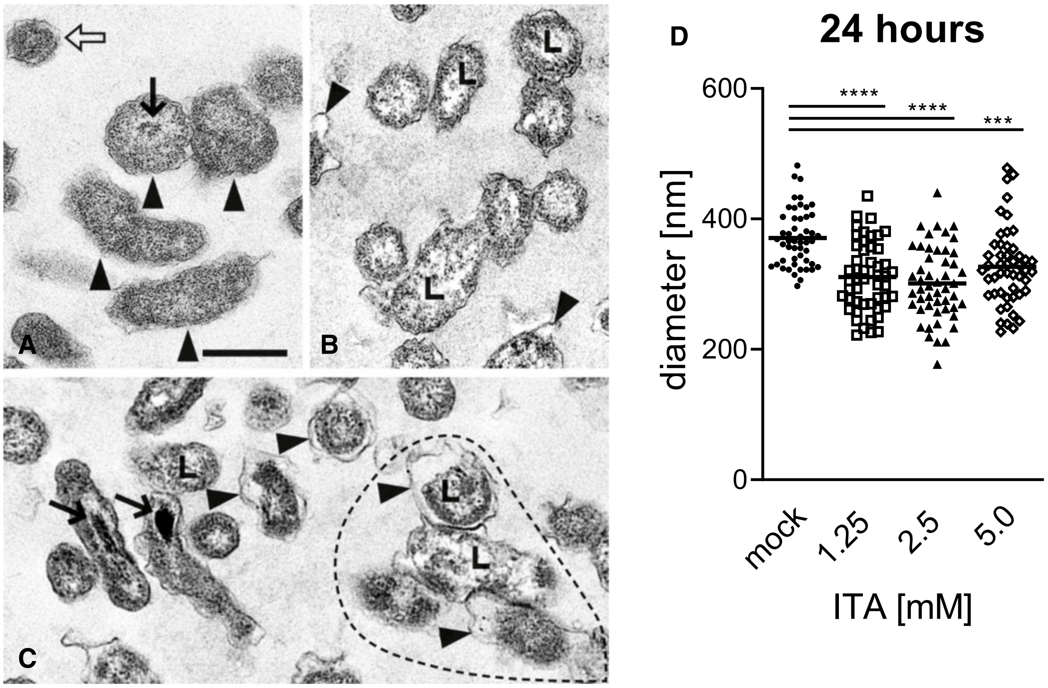

Figure EV4. Ultrastructure of *Coxiella burnetii* NMII from axenic cultures after 24 h.

A–C (A) without ITA: Normal morphology of four LCV (arrowheads) and one SCV (open arrow). Note centrally located nucleoid (thin arrow). (B) with 1.25 mM ITA: LCV with unevenly distributed cytoplasm, clumped at the cytoplasmic membrane and lytic centers (L, examples). Some LCV have dilated periplasmic spaces (arrowheads). (C) with 5.0 mM ITA: LCV have markedly reduced diameters compared with controls in (A) and dilated periplasmic spaces (arrowheads). Their cytoplasm is clumped and centers are lytic as in (B) (L, examples). Four distorted LCV form an aggregate (surrounded by hatched line). Two LCV have electron dense calcium deposits in the area of the nucleoid (arrows). (A), (B), (C) are of the same magnification, scale bar = 500 nm.

D Quantification of LCV diameters after treatment of *C. burnetii* NMII with ITA. Images from electron microscopy were analyzed by random selection of 50 bacterial LCV for measurement of the cell diameter. Each dot represents one cell, mean values are indicated. Statistical analysis was done by One-way ANOVA, with comparison to the mock condition, followed by Dunnett's correction for multiple testing. Asterisks indicate $P = 0.0001$ (***) and $P < 0.0001$ (****).

 © 2022 The Authors

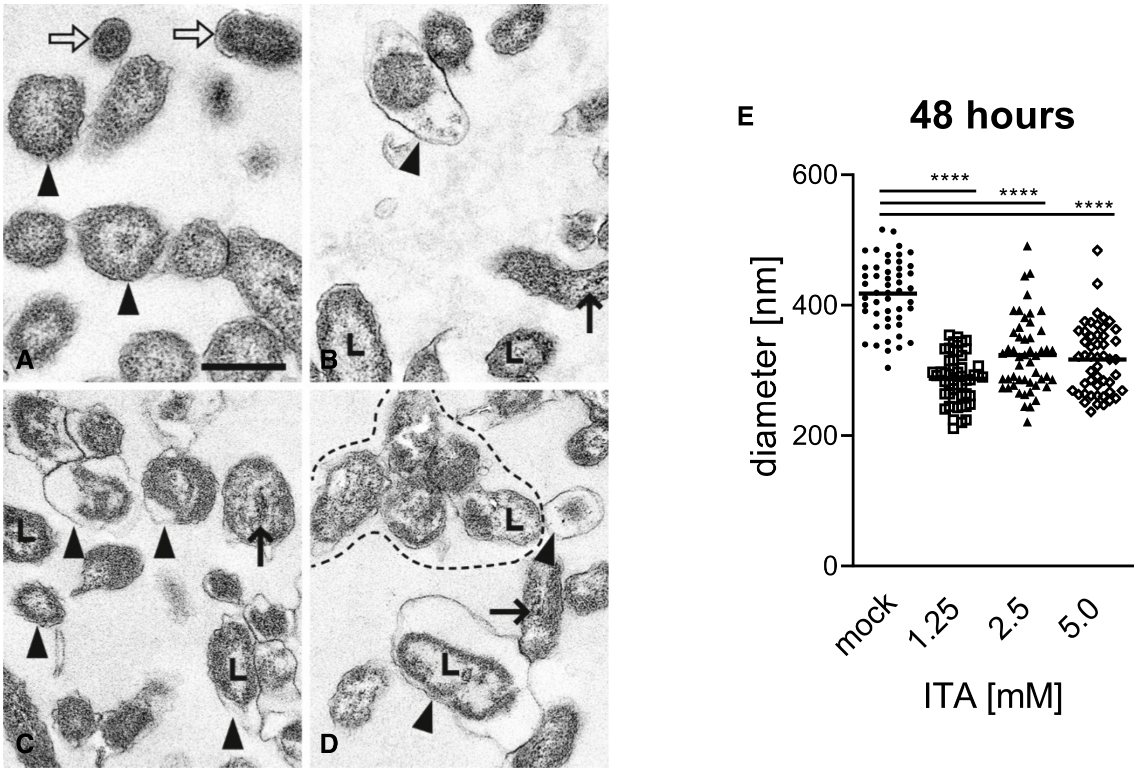

Figure EV5. Ultrastructure of *Coxiella burnetii* NMII from axenic cultures after 48 h.

A–D (A) without ITA: Normal morphology of several LCV (arrowheads, examples) and two SCV (open arrows). (B) with 1.25 mM ITA: LCV with unevenly distributed cytoplasm, clumped at the cytoplasmic membrane and lytic centers (L). One LCV is markedly shrunken and has a severely dilated periplasmic space (arrowhead). Electron dense calcium deposits in the area of a nucleoid (thin arrow). (C) with 2.5 mM ITA: Many shrunken LCV with dilated periplasmic spaces (arrowheads). Some have lytic centers (L), others electron dense calcium deposits in the area of the nucleoid (thin arrow). (D) with 5.0 mM ITA: Shrunken LCV with dilated periplasmic spaces (arrowheads). Some have lytic centers (L), others electron dense calcium deposits in the area of the nucleoid (thin arrow). Distorted LCV form an aggregate (surrounded by hatched line). (A), (B), (C), (D) are of the same magnification, scale bar = 500 nm.

E Quantification of LCV diameters after treatment of *C. burnetii* NMII with ITA. Images from electron microscopy were analyzed by random selection of 50 bacterial LCV for measurement of the cell diameter. Each dot represents one cell, mean values are indicated. Statistical analysis was done by One-way ANOVA, with comparison to the mock condition, followed by Dunnett's correction for multiple testing. Asterisks indicate $P < 0.0001$ (****).
