# Supplementary material for: Macrophages inhibit Coxiella burnetii by the ACOD1‐itaconate pathway for containment of Q fever
Source: EMBO Mol Med. 2022 Dec 7;15(2):e15931. doi: 10.15252/emmm.202215931 (PMC9906395; doi:10.15252/emmm.202215931)
Supplement: Supplementary file 3 [file EMMM-15-e15931-s002.pdf]

EMBO Molecular Medicine

Macrophages inhibit Coxiella burnetii by the ACOD1-itaconate pathway for containment of Q fever

Lisa Kohl, Md. Nur A Alam Siddique, Barbara Bodendorfert, Raffaela Berger, Annica Preikschat, Christoph Daniel, Martha Ölke, Elisabeth Liebler-Tenorio, Jan Schulze-Luehrmann, Michael Mauermeir, Kai-Ting Yang, Inaya Hayek, Manuela Szperlinski, Jennifer Andrack, Ulrike Schleicher, Aline Bozec, Gerhard Krönke, Peter Murray, Stefan Wirtz, Masahiro Yamamoto, Valentin Schatz, Jonathan Jantsch, Peter Oefner, Daniel Degrandi, Klaus Pfeffer, Katja Mertens-Scholz, Simon Rauber, Christian Bogdan, Katja Dettmer, Anja Lührmann, and Roland Lang
DOI: 10.15252/emmm.202215931

Corresponding author: Roland Lang (roland.lang@uk-erlangen.de)

Review Timeline:

Submission Date:	22nd Feb 22
Editorial Decision:	23rd Mar 22
Revision Received:	22nd Oct 22
Editorial Decision:	15th Nov 22
Revision Received:	19th Nov 22
Accepted:	22nd Nov 22

Editor: Zeljko Durdevic

Transaction Report:

(Note: With the exception of the correction of typographical or spelling errors that could be a source of ambiguity, letters and reports are not edited. Depending on transfer agreements, referee reports obtained elsewhere may or may not be included in this compilation. Referee reports are anonymous unless the Referee chooses to sign their reports.)

23rd Mar 2022

Dear Prof. Lang,

Thank you for the submission of your manuscript to EMBO Molecular Medicine. We have now received feedback from the three reviewers who agreed to evaluate your manuscript. As you will see from the reports below, the referees acknowledge the interest of the study but also raise important concerns that should be addressed in a major revision. Focus of the revision should be on the experiments with Coxiella burnetii virulent strain using a relevant intratracheal model and in providing mechanistic insight of itaconate-mediated inhibition of Coxiella replication.

Further consideration of a revision that addresses reviewers' concerns in full will entail a second round of review. EMBO Molecular Medicine encourages a single round of revision only and therefore, acceptance or rejection of the manuscript will depend on the completeness of your responses included in the next, final version of the manuscript. For this reason, and to save you from any frustrations in the end, I would strongly advise against returning an incomplete revision.

We would welcome the submission of a revised version within six months for further consideration. Please let us know if you require longer to complete the revision.

I look forward to receiving your revised manuscript.

Yours sincerely,

Zeljko Durdevic

Zeljko Durdevic
Editor
EMBO Molecular Medicine

***** Reviewer's comments *****

Referee #1 (Remarks for Author):

Kohl et al. describe ACOD1/IRG1-derived itaconate as a crucial macrophage-intrinsic effector molecule for controlling Coxiella infection in vitro and in vivo, and suggest that itaconate could be used as a treatment strategy for chronic Q fever. The study is well conducted and the results are interesting. I have only few comments:

1) It would be interesting to further investigate the anti-inflammatory effect of itaconate in this infection model. For example, could the authors investigate cytokine production and immune cell recruitment at an early time point when the bacterial load is not yet different (perhaps even before day 7)? Is there any indication for enhanced tissue damage in Acod1-/- mice?
2) Can the authors speculate on the mechanism of how itaconate acts on Coxiella?

Referee #2 (Comments on Novelty/Model System for Author):

The model system and relevance is commented upon in the response to the authors. Regarding stats, I was puzzled about all the different methods used in the various figures (except when not listed) - but I did not really check if this was correct or should be changed. It should be done if the paper is considered.

Referee #2 (Remarks for Author):

The study by Kohl et al shows that ACOD1, a metabolic enzyme converting cis-aconitate to itaconate, is part of the host defense controlling early phases of Coxiella burnetii (Cox) infection in mice by restricting its replication within macrophages. The study picks up on previous studies by the group where they found that MyD88 is required for mediating early protection, possibly by contributing to protective IFNgamma and ISGs like iNOS, GBPs. In the present study, Kohl et al follow up these findings by investigating some of the ISGs that has been shown to be protective either in other studies of Q-fever/Cox, or for other intracellular infections. They find that only ACOD1/IRG1, and not iNOS, GBPs or IDO, is important for controlling Cox replication in macrophages, and that itaconate can restore pathogen control in macrophages and in vivo in ACOD1 deficient mice. The protective effect of ACOD1-itaconate has been shown for an increasing number of infections lately, and the current study does not provide any additional mechanistic insight, including on how itaconate inhibits Cox replication.

Also, the conclusion that "ACOD1-derived itaconate ... may be exploited for novel therapeutic approaches in chronic Q fever" is a bit exaggerated: the study shows that itaconate injections can restore control in ACOD1 KOs (day 7) but does not explore if itaconate improves infection/Q-fever in wild-type animals. In addition, since the infection resolves by day 11, it does not link ACOD1 to development of chronic Q fever.

The study is convincing in that ACOD1/itaconate contributes to early control of Cox, but the phenotype is transient (only day 7 post infection in the most relevant intratracheal infection model) - even in ACOD1 deficient animals, the infection is self-limiting and does not spread. The authors suggest this could be due to e.g., IFNgamma and adaptive immunity being induced since INFgamma can rescue the ACOD1 KO phenotype in macrophages. However, the mechanism is not explored, and initial experiments with iNOS and GBP KOs show these are not involved?

Comments:
- In general, it is not clear why the authors choose to include the i.p. mouse infection model when they do have and use a more

relevant intratracheal infection model.
- More information about the experiments would make the results section easier to read, not only in the legends. E.g., the actual time points of infection shown in the figures.
- Gene equivalents are fine and probably validated. But it would be nice to see CFU and/or fluorescence quantifications of bacterial loads in several Figures.
- It seems like 3 independent mouse infection experiments are done for the day 7 time point, with a total number of 12/13 mice pooled (thus 4-5 per exp, repeated three times - which is good) whereas the day 11 time point is only done once with 4/5 mice/group. This is a concern with regards to conclusions about the day 11 vs day 7 time points.

Figure 1:
1a - These gene expression analyses were from experiments done in the previous study (i.p. infection). It would be interesting to see similar gene expression profiles from lungs of intratracheally infected mice.
1b, c - what are data normalized against - the average value of uninfected cells at 4h p.i? Do ACOD1 expression decrease over time in culture and equally much in uninfected and infected cells?
1c - two donors, triplicate wells and one exp. shown. Hard to see 3 dots per time point - are all time points in triplicates but fully identical at some time points (e.g, medium @20h)? Ideally, three three biological replicates should be shown.
1d - 7 mice for TNF-/- and IFNAR-/- vs more for the other genotypes could explain the larger variation for TNF-/- and IFNAR-/- vs MyD88-/- and thus statistics?
Lacks information on how statistical analyses were performed - including in the methods section (refer to figure legends).

Figure 2:
2a - it would help the reader to show how NMII multiplies over time to appreciate the contribution of ACOD1. It would have been interesting to include Myd88-/- as a positive control for the experiment, especially since the deletion of IDO did not show a phenotype.
2b - indication of CCV - should show how these are defined by showing brightfield or at least outline individual cells. Images are nice, but quantifications of bacterial load would be more convincing. Why stain for Cox when you have tdTomato-strain available?
2c, d - errorbars on duplicate measurements (technical) from two donors (biological) are misleading even if stated in the legend. line 183 " ..corresponding to strongly enlarged CCVs filled with bacteria (Fig. 2C)." Is this based on staining in 2b? Should show images of macrophages infected with the tdTomato-strain and quantify.
2d - 72h time point missing for ACOD-/-? Also, IFNg effect suggests that ACOD1 is only relevant in the absence of IFNg. Mechanism for IFNgamma protection?

Fig S1 - what is going on with DAPI staining - are CCVs full of DNA? Impossible to make sense of LAMP1 vs nuclei vs CCV? Nuclear disintegration in ACOD1 KOs?

Figure 3:
Why are experiments sometimes done with Bl6 WTs and other times ACOD+/- hets as controls? Text says WTs are used in Fig 3A, B - but Figures indicate hets are used?
3B - how can ACOD1 KO spleens eliminate Cox compare to WTs at day 11? Perhaps not significant, but this is most likely due to a couple of the mice having cleared the infection from the spleen?
3C - since the phenotype was transient, it would be good to see tissue sections from day 11 as well to verify the GE data. Similar for the other organs, it is difficult to say how much cox is spreading to liver and spleen and thus interpret organ bacterial loads from GE values. Image to the left says ACOD+/+ - should be +/-.
- Since in vitro experiments suggest this is a cell-intrinsic mechanism of controlling the infection - do BMDMs represent some specific cell-intrinsic mechanism found in lung macs - but not spleen and liver macs?
3D - I.p. infection, even if showing that systemic infection gives a phenotype, one can hardly argue that this is more relevant than intratracheal infection since the authors say initially that the way to get Q fever is from inhalation? What happens at other time points after i.p infection, before /after day 7? Does the infection resolve?
3F - there is a significant difference in liver ACOD1-/- receiving het bone-marrow, despite written in the text that there is no contribution outside the haemopoietic compartment.

It would be interesting to see the relative cell populations in the lungs after intratracheal infection in het/WT vs ACOD1 KO mice. Anything like what Nair et al. shows for Mtb (PMN infiltration)?

Figure 4:
4B - why were i.p infections terminated at day 7, including bacterial burden (Fig 3)? It makes the data less informative with regards to comparing results from the two models- also for resolution.
4D, E - why are the mice recovering at day 11 if not from some induced protective immune response, and what could that be - since e.g., IFNgamma is lower in the lungs and spleen at day 11 post infection when the infection resolves? Cytokine levels should preferentially be shown as protein levels - relative gene-expression cannot be used to explain biological effects. Especially since it is shown that IFNgamma can rescue ACOD1 deficiency, it would be relevant to see IFNgamma protein levels in the organs of infected mice during the 11 days of infection to see if this could explain resolution.

Figure 5:
5A - using technical replicate to calculate significance is not a good practice.
5D - should ideally do siRNA ACOD1 to demonstrate that ACOD1 is needed in MDMs.
5I/J - These data are convincing. However, the experiments should have included WT+ itaconate to check if the treatment effectively lowers the burden of WT mice - to justify the treatment suggestion. As for now, only rescue of the ACOD1 KO is shown. Also, the experiments would be more relevant if done with the intratracheal infection model - and continued to day 11 post infection, measuring both weight over time and organ load at day 7 and 11. Tissue images with quantification is appreciated - and should be used more to complement GE (which is more difficult to interpret), and for all organs.
The legend (or figure) does not inform the time point of the results, but assuming it is day 7.

Minor comments:
Line 136: Michelucci et al have shown that itaconate restrict TB growth on axenic culture, not in cells.
Line 137: the authors do not mention the work of Hall et al showing that IRG1 is needed to control Salmonella in a Zebra Fish model (Cell Metabolism, 2013), Nair et al (JEM, 2018) showing that IRG1 is needed to restrict TB in a mouse model, the work of Daniels et al showing that IRG1 and 4-OI are needed to control Zika virus infection in a mouse model (Immunity, 2019).
Figure 1a legend: Figure shows 5h and 20h post infection but 20h is not mention in the legend
Line 201 - says significantly higher load - but not where? Figures show it is in the KOs, but this should be spelled out in the text.
Line 219 - "attenuated weight loss" - meaning? Sentence is nested and it is difficult in the end to interpret what is really communicated - that organ bacterial load correlates with inflammatory gene expression but not necessarily with pathology (weight loss) - or the other way around?
Line 222-223 - GBPs and NOS are not inflammatory cytokines
Line 366 - claim IFNgamma is high at day 7 and 11. No measurements of that?
The discussion opens with saying ACOD1 is essential - this can hardly be true, since the infection is self-limiting, and no mice die? Major protective mechanism is ok.

Referee #3 (Comments on Novelty/Model System for Author):

Model is adequate, but strengths and weaknesses should be added to discussion. Given that these results need to be confirmed with virulent bacteria, the medical impact is medium.

Referee #3 (Remarks for Author):

In this manuscript, Kohl et al explore the role of nutritional immunity, specifically citrate metabolism, during Coxiella infection. The mammalian enzyme ACOD1 decarboxylates cis-aconitate to itaconate (ITA); recent evidence for numerous bacterial pathogens indicates that ACOD1 and ITA restrict pathogen growth through various mechanisms. This group recently established a new mouse model for Coxiella infection, using myd88-deficient mice and avirulent Coxiella NMII. In this study, they found that ACOD1 is upregulated shortly after Coxiella infection in both the mouse as well as in vitro in mouse bone marrow-derived macrophages and primary human monocyte-derived macrophages. Coxiella infection improves in ACOD1-knockout macrophages and mice. Important points include that ACOD1 expression in hematopoietic cells is critical and that exogenous ITA inhibits Coxiella growth. Finally, ITA directly inhibits Coxiella in axenic media, though an immunomodulatory role in macrophage cannot be ruled out.

Overall, this is a very nice study, clearly and logically written with an impressive balance of in vitro and in vivo experiments. The experiments are well done, and conclusions are based on multiple lines of evidence and in general not over-interpreted. While performed with avirulent bacteria, the authors have uncovered a potentially critical component of the host immune response to Coxiella infection. While not necessarily novel for the broader field of bacterial pathogenesis, nutritional immunity in the context of Coxiella infection is relatively unexplored and is therefore of significance and high impact.

Major comments:

1) This study uses an immune-deficient mouse model (myd88 KO) as well as avirulent bacteria. While the data indicating that ITA directly inhibits Coxiella growth in axenic media helps alleviate concerns that the ACOD1/ITA finding is an artifact of the model system, the discussion should address the strengths and weaknesses of this model, and that these findings need to be confirmed with wildtype mice and bacteria (NMI).

2) For in vitro infections of mutant cells, did the authors confirm that the initial infection levels (day 0) were similar?

Minor comments

1. In many cases, the figure legends need to clarify how samples were normalized when comparing gene expression. For example, it's not clear that in figure 4 D-F that expression was first normalized to naïve/uninfected mice.
2. Figure 5F, which shows growth in axenic media with different concentrations of ITA, should be shown as GE or CFU, which is

the norm in the field. It is difficult to determine the extent of the growth defect (e.g., one log), when shown as OD.

Point-by-point reply to the comments of the Editor and of the Reviewers

Editor's comments:

The referees acknowledge the interest of the study but also raise important concerns that should be addressed in a major revision.

Response: We thank the Editor and the Reviewers for a thorough and comprehensive critical review of our manuscript, for the overall positive evaluation, and for the invitation to submit a revised manuscript. As detailed in the point-by-point reply to the Reviewers' comments below, we have carefully considered each comment and criticism. We performed several new experiments to address the questions raised by the Reviewers and the Editor.

Focus of the revision should be on the experiments with *Coxiella burnetii* virulent strain using a relevant intratracheal model and in providing mechanistic insight of itaconate-mediated inhibition of Coxiella replication.

Response:

- Experiments with virulent *C. burnetii*:

We agree that it is highly desirable to validate the important role of ACOD1 and itaconate found by us using the C. burnetii *Nine Mile Phase II strain in experiments using a virulent strain. However, there are severe logistical obstacles to experiments with virulent* C. burnetii*, especially with regard to* in vivo *infections. Virulent* C. burnetii *Nine Mile Phase I is a BSL-3 organism that can only be handled in specialized labs with specific permission by the regulatory authorities. Only few labs in the world have the possibility to perform* in vivo *infections with virulent* C. burnetii*. Currently, there is no such laboratory in Germany. We are in the process of setting up a collaboration with a lab with BSL-3 animal facilities for* in vivo *analysis of virulent* C. burnetii *in the future. However, this will require substantially more time to prepare and organize than available for this manuscript revision.*

In discussion of these issues with the Editor, it was agreed that for the manuscript revision we will perform in vitro *experiments with virulent* C. burnetii *Nine Mile Phase I (NMI) to determine whether key findings made using the NMII strain can be replicated for the virulent NMI strain. Fortunately, we could collaborate with the group of Dr. Katja Mertens-Scholz (Friedrich Löffler Institute, Jena, Germany) who runs an active research program on virulent* C. burnetii *and provided the access to their BSL-3 laboratory.*

Thus, we could address two key questions: (1) does itaconate inhibit directly the growth of virulent C. burnetii *in axenic culture? And (2) is the inhibition of* C. burnetii *growth in macrophages by itaconate also observed for the virulent strain?*

Ad (1) We demonstrate in the revised manuscript that itaconate inhibits growth of the virulent strain in axenic culture in a dose-dependent manner, very similar to the results obtained with the attenuated NMII strain. While lower concentrations between 0.3 and 1.25 mM already inhibited replication when added at the start of the cultures, higher doses of 2.5 to 5 mM strongly reduced CFU numbers even when added to an established culture on day 5. These new results are now included as Fig. 5K and 5L in the revised manuscript.

Fig. 5K + L in revised manuscript

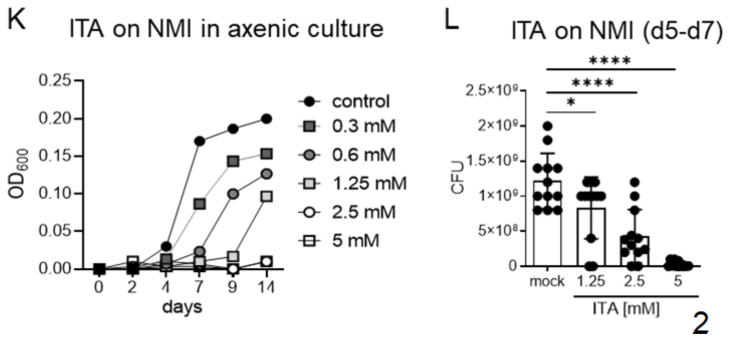

Ad (2) To examine whether exogenous itaconate can inhibit also the growth of virulent NMI in macrophages, we employed the human monocytic cell line THP-1 (differentiated to macrophages with PMA) for in vitro *infection and treatment with itaconate. Virulent* C. burnetii *replicated efficiently in THP-1 cells, but intracellular growth was efficiently suppressed by itaconate at a concentration of 2.5 mM (see new Fig. 5M in the revised manuscript). These data are very similar to what we observed for the attenuated* C. burnetii *NMII in human monocyte-derived macrophages.*

Thus, itaconate treatment exerts a direct inhibitory effect on the replication of virulent C. burnetii *in axenic cultures and when added to infected macrophages at the same concentrations as for the attenuated NMII. This direct growth inhibition by itaconate is most likely the basis for the phenotype of ACOD1 knockout mice and the effective reduction of* C. burnetii *NMII growth by treatment of these mice with itaconate. Therefore, it is reasonable to expect that ACOD1-itaconate will also be active during infection with virulent* C. burnetii in vivo*.*

Fig. 5M

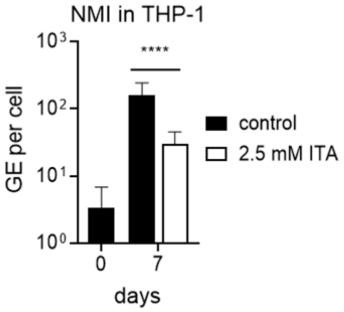

- Mechanistic insight of itaconate-mediated inhibition of Coxiella replication:

At present, the mode of action for inhibition of C. burnetii *growth by ITA is unknown. To address this question rigorously and in detail will constitute a new, separate project. Here, we have performed several experiments to obtain new insights into how itaconate treatment acts on* C. burnetii*.*

First, as described above, we confirmed that virulent Phase I C. burnetii *are inhibited by itaconate similar to the NMII strain. These findings indicate the mechanism of itaconate-mediated inhibition does not involve the specific lipopolysaccharide structures in the cell wall of* C. burnetii*.*

Second, delayed addition of itaconate to established cultures of Phase I and Phase II C. burnetii *showed a bactericidal effect at the high concentration of 5 mM (which can be reached in murine macrophages through the activity of ACOD1).*

Third, electron-microscopic studies were performed in collaboration with Dr. Elisabeth Tenorio-Liebler to assess the direct effect of titrated concentrations of itaconate on the ultrastructural morphology of C. burnetii*. These analyses revealed shrinkage of the cell body causing dilation of the periplasmic space between the cytoplasmic membrane and the outer cell membrane. The center of bacteria treated with itaconate was lytic and deposits of electron dense calcium apatite crystals at the fibrils of the nucleoid were observed. Such calcium deposits are a frequent finding in degenerate and necrotic cells and tissues, and suggest death of the* C. burnetii *cells. In addition, at the 48 h timepoint, many cells in the preparation had the diameter of the small cell variant (SCV) form, suggesting that itaconate may induce a transition to the non-replicating form in some of the bacterial cells.*

Fourth, to follow-up this observation, we performed qRT-PCR to assess the expression of dormancy-associated genes in itaconate-treated C. burnetii*. Although we observed a dose-dependent induction of the expression of Scva, a gene specific for the SCV form of* C. burnetii*, in one experiment, this could not be replicated in two additional experiments.*

The findings of shrinkage of bacterial cells, dilation of the periplasmic space and indications of bacterial cell death made in the ultrastructural analysis are now included in the Results section of the manuscript and shown as Expanded View Figures EV4+5.Together, these new data on the direct action of itaconate on C. burnetii *provide an ultrastructural correlate of the observed bactericidal effect and will serve as a starting point to investigate the underlying mechanisms in future work.*

Reviewer's comments

Referee #1 (Remarks for Author):

Kohl et al. describe ACOD1/IRG1-derived itaconate as a crucial macrophage-intrinsic effector molecule for controlling Coxiella infection in vitro and in vivo, and suggest that itaconate could be used as a treatment strategy for chronic Q fever. The study is well conducted and the results are interesting. I have only few comments:

1) It would be interesting to further investigate the anti-inflammatory effect of itaconate in this infection model. For example, could the authors investigate cytokine production and immune cell recruitment at an early time point when the bacterial load is not yet different (perhaps even before day 7)?

Response: This is an interesting question, given the many reports suggesting that ACOD1-itaconate downregulates inflammation. As the Reviewer suggests, it should best be analyzed under conditions when bacterial burden is not different to avoid secondary effects on inflammatory gene expression. The original manuscript contains gene expression data for Il6, Ifng, Nos2 and Gbp1 from the spleen of mice after intratracheal infection, when bacterial burden is not significantly different at d7 (Fig. 4C in revised manuscript vs. Fig. 3A). These show a moderate increase in mRNA expression in Acod1$^{-/-}$ spleens, consistent with a regulatory function of endogenous ITA production.

As suggested by the Reviewer, we have now infected Acod1$^{-/-}$ and Acod1$^{+/-}$ mice intratracheally and analyzed bacterial burden, cytokine expression and immune cell recruitment in the lung on day 2 after infection (Response Fig. 1). Indeed, at this time point the bacterial burden assessed as GE by PCR was not different. By qRT-PCR for cytokines, induction of IL-6 and and Gbp1 was observed, but not increased in Acod1$^{-/-}$, whereas IFNγ, Nos2 and CD3 mRNAs were not different to controls, suggesting that T cells are not recruited yet at this early timepoint. By Legendplex analysis of cytokine protein levels in lung homogenates, we observed a trend for increased production of IL-1α and TNF in Acod1$^{-/-}$ mice. By flow cytometry, we observed a reduction in the frequency of SiglecF^{+} CD11c^{+} alveolar macrophages after infection that was accompanied by a slight increase in the fraction of monocytes and neutrophils. Altogether, the effects of Acod1-deficiency on bacterial burden, the early inflammatory response and cell recruitment at this early time point (day 2) were not very strong.

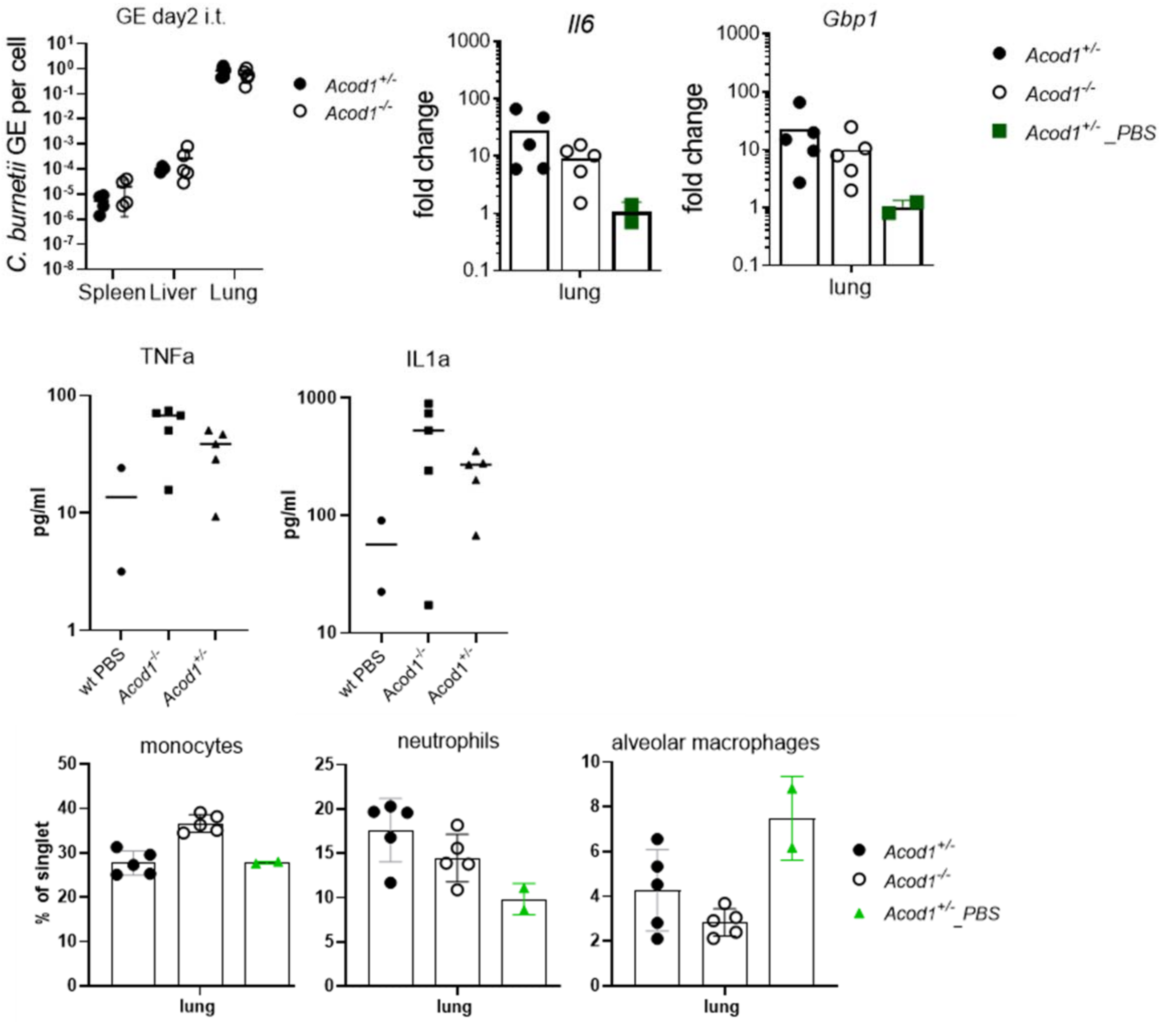

Response Fig. 1: bacterial burden, gene expression, cytokine protein and cell recruitment in the lung day 2 after i.t. infection.

We also analyzed these parameters later in infection on day 11, when bacterial burden was again comparable between genotypes (Fig. 3B). While Gbp1 mRNA was higher (Fig. 4D), and protein levels of IFNγ and IL-1a showed a trend for an increase in Acod1$^{-/-}$ (Response Fig. 2), no evidence for enhanced cellular infiltration by neutrophils or monocytes was observed by flow cytometry.

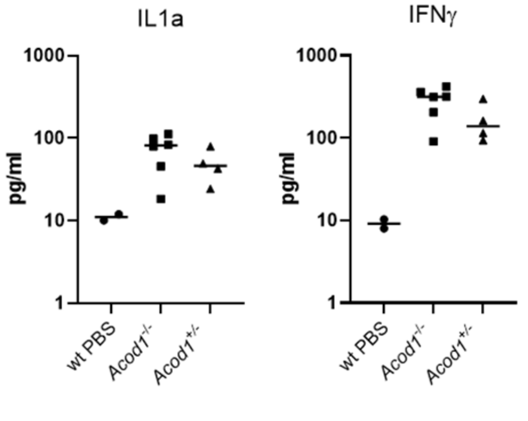

Response Fig. 2: cytokine proteins in the lung day 11 after i.t. infection.

Is there any indication for enhanced tissue damage in Acod1-/- mice?

Response: *Increased cytokine expression and inflammation may indeed cause tissue damage. In preliminary experiments, we have observed that protein levels of the alarmin cytokine IL-1α were not only increased on day 2 after i.t. infection (see paragraph above) but also on day 11 after i.p. infection in the livers of Acod1$^{-/-}$. To determine the extent of cell death in tissues, Caspase-3-positive cells were stained in the lungs and livers on day 7 after i.t. and i.p. infection, respectively, but no difference between genotypes was found (Response Fig. 3).*

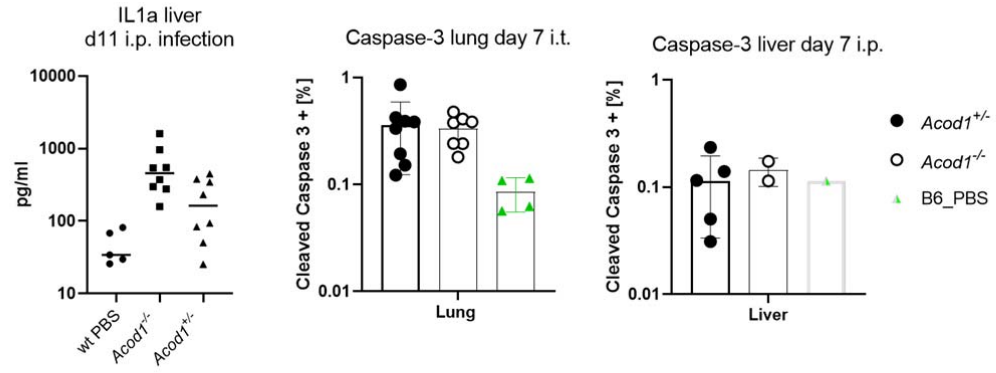

Response Fig. 3: IL-1a and Caspase-3+ cells in lung and liver.

Thus, while ACOD1-deficient mice may develop increased immunopathology and tissue damage during C. burnetii *infection, we have not yet systematically addressed this question. Doing so will require a comprehensive and careful analysis of histopathological changes, as well as measurement of markers for cell death and tissue function, from different organs at different stages after infection. Such a comprehensive investigation was beyond the scope of the revision of this manuscript.*

2) Can the authors speculate on the mechanism of how itaconate acts on Coxiella?

Response: *please see response to Editor's comments on page 2 of this point-by-point reply*.

Referee #2 (Comments on Novelty/Model System for Author):

The model system and relevance is commented upon in the response to the authors. Regarding stats, I was puzzled about all the different methods used in the various figures (except when not listed) - but I did not really check if this was correct or should be changed. It should be done if the paper is considered.

Response: *statistical testing was performed using GraphPad Prism software. The choice of different tests was dependent on the data structure (e.g. normal distribution of data) and type of comparisons. The description in the methods has been edited to clarify the procedure. In addition, in each figure legend the type of test performed is given.*

Referee #2 (Remarks for Author):

The study by Kohl et al shows that ACOD1, a metabolic enzyme converting cis-aconitate to itaconate, is part of the host defense controlling early phases of Coxiella burnetii (Cox) infection in mice by restricting its replication within macrophages. The study picks up on previous studies by the group where they found that MyD88 is required for mediating early protection, possibly by contributing to protective IFNgamma and ISGs like iNOS, GBPs. In the present study, Kohl et al follow up these findings by investigating some of the ISGs that has been shown to be protective either in other studies of Q-fever/Cox, or for other intracellular infections. They find that only ACOD1/IRG1, and not iNOS, GBPs or IDO, is important for controlling Cox replication in macrophages, and that itaconate can restore pathogen control in macrophages and in vivo in ACOD1 deficient mice. The protective effect of ACOD1-itaconate has been shown for an increasing number of infections lately, and the current study does not provide any additional mechanistic insight, including on how itaconate inhibits Cox replication.

<u>Response:</u> *The induction of ACOD1-itaconate indeed has received considerable attention as a defense mechanism in different infections and we have referred to these studies in our manuscript. While it is established that ACOD1-itaconate do play a role in host defense, many questions remain open, including (1) which pathogens are inhibited by itaconate, (2) whether itaconate acts directly on microbial replication or indirectly via changes in the host cell, and (3) whether effects observed* in vitro *also occur* in vivo*. Thus, much work remains to be done to elucidate the function of ACOD1-itaconate in host defense during infection.*

This certainly applies to the specific case of Q-fever caused by C. burnetii*: to date, a Pubmed search for “Coxiella” or “Q fever” and “itaconate” or “Acod1” does not produce a single hit; despite the demonstration that IFNγ is required for restriction of* C. burnetii *in macrophages already 15 years ago, it has remained largely enigmatic how macrophages control the growth of these intracellular bacteria. Therefore, the demonstration that ACOD1-itaconate is a major protective mechanism for controlling* C. burnetii *in macrophages and* in vivo *is both original and relevant.*

Also, the conclusion that "ACOD1-derived itaconate ... may be exploited for novel therapeutic approaches in chronic Q fever" is a bit exaggerated: the study shows that itaconate injections can restore control in ACOD1 KOs (day 7) but does not explore if itaconate improves infection/Q-fever in wild-type animals. In addition, since the infection resolves by day 11, it does not link ACOD1 to development of chronic Q fever.

<u>Response:</u> *The Reviewer correctly points out that itaconate was used by us for treatment of Acod1$^{-/-}$ mice* in vivo*, where it effectively reduced bacterial burden to the levels observed in WT mice. For interpreting these results, it is important to consider that WT mice and macrophages efficiently inhibit replication of* C. burnetii*, and they generate high levels of itaconate.*

Addition of exogenous itaconate to infected WT macrophages did not further reduce C. burnetii *load* in vitro *(Fig. 5B). As suggested by the Reviewer, we have performed a preliminary experiment of treatment with itaconate in infected Acod1$^{+/-}$ mice and found that it did not further reduce* C. burnetii *burden (Response Fig. 4, see also response to the Reviewer’s comment to Fig. 5I/J below).*

Response Fig. 4: treatment of wild-type mice (*Acod1$^{+/-}$* or *Acod1$^{+/+}$*) with itaconate (daily injection of 1 mg ITA i.p.) after i.p. infection with NMII.

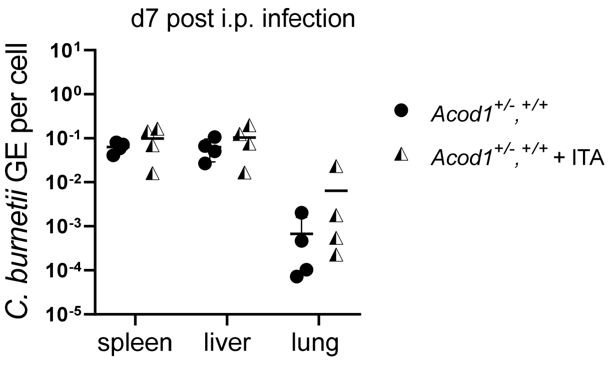

Given that WT mice generate ample itaconate and control infection rapidly, this finding was not surprising. Does this argue against the potential of itaconate as a treatment strategy for Q fever? Human macrophages are very weak producers of itaconate, as we found in GC-MS analysis that are included in the revised manuscript (Fig. EV3); C. burnetii *replicate well in primary human macrophages (Fig. 5D); treatment of human macrophages with itaconate inhibits* C. burnetii *replication (Fig. 5E); this also holds true for human THP-1 macrophages infected with the virulent strain* C. burnetii *Nine Mile Phase I (Fig. 5M, see below). In conclusion, based on the species differences in production of itaconate and the efficacy of exogenous itaconate in human macrophages, we sustain our conclusion that it "may be exploited for novel therapeutic approaches in chronic Q fever".*

The study is convincing in that ACOD1/itaconate contributes to early control of Cox, but the phenotype is transient (only day 7 post infection in the most relevant intratracheal infection model) - even in ACOD1 deficient animals, the infection is self-limiting and does not spread. The authors suggest this could be due to e.g., IFNgamma and adaptive immunity being induced since INFgamma can rescue the ACOD1 KO phenotype in macrophages. However, the mechanism is not explored, and initial experiments with iNOS and GBP KOs show these are not involved?

Response: *The Reviewer rightly points out that we do not explore the ACOD1-independent mechanisms by which IFNγ inhibits replication of* C. burnetii *in macrophages or after d7* in vivo*. In preliminary data, we observed that treatment with IFNγ still led to inhibition of* C. burnetii *growth in Ido1/2$^{-/-}$, Nos2$^{-/-}$ and Gbp2$^{-/-}$ BMM (not shown in the manuscript). Which of the many IFNγ-induced genes is really essential for control of* C. burnetii in vivo *in the later phase is an important question that will be studied and hopefully resolved in future experiments.*

Comments:
- In general, it is not clear why the authors choose to include the i.p. mouse infection model when they do have and use a more relevant intratracheal infection model.

Response: *We agree that the i.t. model is better mimicking the natural infection.*

The i.p. model shows the same phenotype with regard to higher bacterial burden in Acod1$^{-/-}$ mice. This was an important finding because it indicates that not only macrophages in the lung require Acod1 for early containment of C. burnetii *(see response to Reviewer's comment to Fig. 3). We now extended the follow-up in the i.p. model to d11 and confirmed that bacterial burden is also reduced at this later time point (new Fig. 3F), consistent with the data after i.t. infection.*

A major advantage of the i.p. model is that it allows treatment with i.p. injections of ITA, which were considered too stressful for the mice after i.t. infection (see response to Reviewer's comment to Fig. 5I/J).

- More information about the experiments would make the results section easier to read, not only in the legends. E.g., the actual time points of infection shown in the figures.

Response: *We thank the Reviewer for this suggestion and have added figure titles to several graphical panels.*

- Gene equivalents are fine and probably validated. But it would be nice to see CFU and/or fluorescence quantifications of bacterial loads in several Figures.

Response: *For measuring* C. burnetii *burden in macrophages and in tissues, we have used mostly determination of gene equivalents by quantitative PCR because it has the big advantage to yield quantitative data and to possess high sensitivity. These are also the reasons why most studies in the* C. burnetii *field employ this method for quantification of bacterial loads in tissues.*

For validation of these results, fluorescent C. burnetii *or immunofluorescence stainings have been used in the* in vitro *experiments to show the phenotype of Acod1$^{-/-}$ BMM (Fig. 2C, D; EV1) and the effect of ITA treatment (Fig. 5C).*

Detection of C. burnetii *in organs from infected mice has been performed by immunohistochemistry using a polyclonal antiserum specific for* C. burnetii*. Data obtained from tissue sections after i.t. and i.p. infection were quantitated by automated image analysis, and are shown in Fig. 3C, E and in Fig. 5G. These data are consistent with the results of qPCR for GE. However, as detailed in the response to the Reviewer's comment to Fig. 5C/G, there are limitations to the sensitivity and quantifiability of IHC.*

CFU determinations to measure viable C. burnetii *were performed to study the direct effect of ITA on the growth of* C. burnetii *in axenic culture (Fig. 5J, new Fig. 5L).*

We have only very recently started to perform cultural analysis for C. burnetii *from mouse tissues. The CFU data obtained to date are promising in that they confirm the impact of knockout for ACOD1 or MyD88 on bacterial growth/persistence observed by qPCR for GE. However, these data are too incomplete and preliminary to include them in this revised manuscript.*

- It seems like 3 independent mouse infection experiments are done for the day 7 time point, with a total number of 12/13 mice pooled (thus 4-5 per exp, repeated three times - which is good) whereas the day 11 time point is only done once with 4/5 mice/group. This is a concern with regards to conclusions about the day 11 vs day 7 time points.

<u>Response:</u> *We have performed an additional intratracheal infection experiment with analysis at d11. Accordingly, the respective Fig. 3B (bacterial burden), Fig. 4A (weight curve) and Fig. 4D (gene expression) in the revised manuscript have been updated to include the results of the repeat experiment. For further description, please see below in response to comments regarding these figures.*

Figure 1:

1a - These gene expression analyses were from experiments done in the previous study (i.p. infection). It would be interesting to see similar gene expression profiles from lungs of intratracheally infected mice.

<u>Response:</u> *We agree with the Reviewer that a similar analysis of gene expression in the lungs of Myd88$^{-/-}$ versus Myd88$^{+/-}$ after intratracheal infection would be interesting. Therefore, we performed one intratracheal infection experiment and sacrificed mice at d7 for bacterial burden and gene expression analysis. While* C. burnetii *GE were significantly higher in Myd88-/- lungs (confirming the results of Kohl et al 2019), we observed a reduced mean expression of IFNγ and Acod1 (Response Fig. 5). Unfortunately, our Myd88 mouse colony did not produce the offspring needed for a repeat experiment. Therefore, we do not mention this preliminary experiment in the manuscript but only in this response to the Reviewer.*

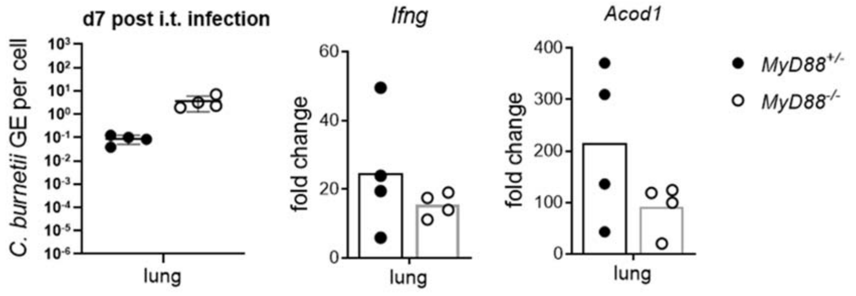

Response Fig. 5: bacterial burden and gene expression in the lungs of Myd88$^{-/-}$ mice on day 7 after intratracheal infection with *C. burnetii* NMII. Data are from one experiment (n=4 mice per genotype).

1b, c - what are data normalized against - the average value of uninfected cells at 4h p.i? Do ACOD1 expression decrease over time in culture and equally much in uninfected and infected cells?

Response: *Ct values of Taqman RT-PCRs for Acod1 were normalized by calculating the ΔCT values versus the housekeeping genes Hprt (mouse, Fig. 1B) or PPIA (human, Fig. 1C). Calibration was performed against average ΔCT values of WT media control samples of the earliest time point (4h in Fig. 1B, 2h in Fig. 1C). This procedure is described in Materials and Methods section. Indeed, it appears that the baseline expression of Acod1 is decreasing over time in non-stimulated cultures of BMM and hMDM.*

1c - two donors, triplicate wells and one exp. shown. Hard to see 3 dots per time point - are all time points in triplicates but fully identical at some time points (e.g, medium @20h)? Ideally, three biological replicates should be shown.

Response: *As suggested by the Reviewer, the experiment was repeated with additional donors. Revised Fig. 1C shows the kinetic results of cells from three donors, each analyzed in triplicate stimulations. For better visual discrimination, data points are now depicted in a staggered manner in Fig. 1B and Fig. 1C.*

1d - 7 mice for TNF-/- and IFNAR-/- vs more for the other genotypes could explain the larger variation for TNF-/- and IFNAR-/- vs MyD88-/- and thus statistics?

Response: *As suggested by the Reviewer, the experiment was repeated with additional $Tnf^{-/-}$ and $Ifnar1^{-/-}$ mice, such that for each genotype at least 9 mice were included. Results are shown as revised Fig. 1D (see below) and confirm the statement from the original manuscript that only for $Myd88^{-/-}$ a significant reduction was observed. The wording of the description in the Results was changed to "TNF and type 1 IFN signaling, …, was less essential" (lines 162-4).*

Lacks information on how statistical analyses were performed - including in the methods section (refer to figure legends).

Response: *One-way ANOVA with Dunnett's multiple comparisons test was performed. This is described now in the revised legend to Fig. 1D; in addition, we expanded the Statistics paragraph in the Methods section.*

Figure 2:

2a - it would help the reader to show how NMII multiplies over time to appreciate the contribution of ACOD1. It would have been interesting to include Myd88-/- as a positive control for the experiment, especially since the deletion of IDO did not show a phenotype.

Response: *The kinetic analysis of* C. burnetii *replication is included in Fig. 2E.*

We have not included the analysis of $Myd88^{-/-}$ BMM because this experiment was done in the Kohl et al 2019 paper already and demonstrated increased replication of C. burnetii *NMII in the knockout BMM.*

2b - indication of CCV - should show how these are defined by showing brightfield or at least outline individual cells. Images are nice, but quantifications of bacterial load would be more convincing. Why stain for Cox when you have tdTomato-strain available?

Response: *The DAPI nuclear staining was used in the immunofluorescence staining for* C. burnetii *in revised Fig. 2C to define individual cells. This experiment revealed the presence of very large CCV in a significant fraction of $Acod1^{-/-}$ BMM. When fluorescent* C. burnetii *expressing tdTomato became available, we repeated the infection of $Acod1^{-/-}$ BMM with this strain, facilitating quantification of bacterial load per cell via the fluorescent tdTomato signal (Fig. 2D). Thus, qPCR*

of GE, immunfluorescence and flow cytometry measurements in Fig. 2A-E complement each other.

2c, d - errorbars on duplicate measurements (technical) from two donors (biological) are misleading even if stated in the legend.

<u>Response:</u> In revised Fig. 2D, we show now the average values of duplicate wells from both experiments; the error bars were removed. For revised Fig. 2E, an additional experiment has been performed, such that now mean and SD of GE are shown for 5 mice per condition.

line 183 " ..corresponding to strongly enlarged CCVs filled with bacteria (Fig. 2C)." Is this based on staining in 2b? Should show images of macrophages infected with the tdTomato-strain and quantify.

<u>Response:</u> We have not used the tdTomato-strain for immunofluorescence analysis. The sentence has been modified to clarify the comparison of the cells displaying the strong tdTomato-signal in flow cytometry shown in Fig. 2D to the immunofluorescent images displayed in Fig. 2C.

2d - 72h time point missing for ACOD-/-? Also, IFNg effect suggests that ACOD1 is only relevant in the absence of IFNg. Mechanism for IFNgamma protection?

<u>Response:</u> We thank the Reviewer for identifying this error in Fig. 2D. The 72h datapoint was inadvertently deleted when introducing the y-axis break. This has been corrected in the revised Fig. 2E. Regarding the control of C. burnetii *replication in IFNγ-treated macrophages: Indeed, as we clearly describe in the manuscript, IFNγ overcomes the deficiency in* C. burnetii *in Acod1$^{-/-}$ BMM. Which of the many IFNγ-induced genes are pivotal to inhibit* C. burnetii *replication is currently unknown. It is likely that very few, if any, will be essential and lead to a severe phenotype if knocked-out in isolation. In preliminary experiments, we did not find enhanced replication in IFNγ-treated BMM deficient in Ido1/2, Gbp2 or Nos2 alone.*

Fig S1 - what is going on with DAPI staining - are CCVs full of DNA? Impossible to make sense of LAMP1 vs nuclei vs CCV? Nuclear disintegration in ACOD1 KOs?

<u>Response:</u> Indeed, in the absence of IFNγ CCVs in Acod1$^{-/-}$ BMM are full of C. burnetii *and the DAPI stains their DNA. In contrast, the penetration of the anti-*C. burnetii *antibody into these large CCVs seems incomplete and the level of LAMP1 staining is much less intense than the DAPI signal. This phenomenon of intense DAPI staining in large* C. burnetii *CCVs was observed previously by us (Hayek 2019 Cell Reports), and we include here a figure from this paper.*

We did not see a clear evidence for nuclear changes in the Acod1$^{-/-}$ BMM after C. burnetii *infection.*

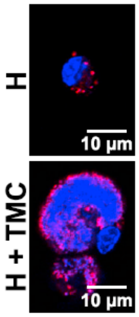

Response Fig. 6: dominant DAPI staining in large CCVs (from Fig. 4F in Hayek 2019 Cell Reports).

Red: C. burnetii-antibody

Blue: DAPI

Figure 3:

Why are experiments sometimes done with Bl6 WTs and other times ACOD+/- hets as controls? Text says WTs are used in Fig 3A, B - but Figures indicate hets are used?

<u>Response:</u> *We apologize for this discrepancy between Figure and Results text description. In fact, we started the experiments using B6 mice as controls. In subsequent experiments, we also used Acod1$^{+/-}$ littermate controls to make sure that the phenotype in Acod1$^{-/-}$ mice was not caused by confounding factors, such as differences in the microbiota between our B6 controls and the Acod1$^{-/-}$ mice. We confirmed the increased* C. burnetii *burden in Acod1$^{-/-}$ mice in comparison to Acod1$^{+/-}$ as well as to B6 controls. Therefore, data from several experiments using B6 or Acod1$^{+/-}$ mice as controls was pooled in Fig. 3. In the revised manuscript, we have corrected the description in the Results text to include "and Acod1$^{+/-}$ animals".*

In addition, we compared BMM from Acod1$^{+/+}$ and Acod1$^{+/-}$ littermates for their capacity to control replication of C. burnetii in vitro. *We did not see a difference in* C. burnetii *GE after 96h of* in vitro *infection (Response Fig. 7), indicating that there is no gene-dosage effect in heterozygous Acod1$^{+/-}$ BMM for control of* C. burnetii *replication.*

Response Fig. 7. *Acod1$^{+/-}$* BMM control *C. burnetii* similar to B6 BMM. Cells were infected with NMII for 4 hours, followed by washing. GE per cell were determined after 96 hours. Each dot represents BMM from one mouse

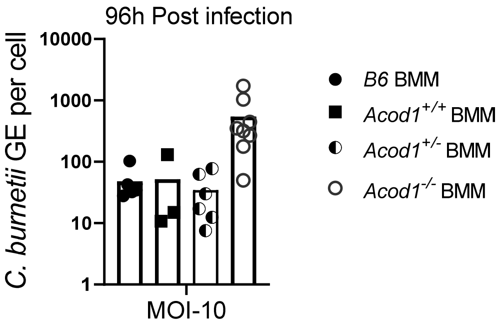

3B - how can ACOD1 KO spleens eliminate Cox compare to WTs at day 11? Perhaps not significant, but this is most likely due to a couple of the mice having cleared the infection from the spleen?

<u>Response:</u> *This experiment has been repeated, such that the revised Fig. 3B shows now results from 11 Acod1$^{-/-}$ and 8 B6/Acod1$^{+/-}$ mice. The bacterial burden in the spleens is low in both genotypes, differences in bacterial load are not significant at this time point.*

3C - since the phenotype was transient, it would be good to see tissue sections from day 11 as well to verify the GE data. Similar for the other organs, it is difficult to say how much cox is spreading to liver and spleen and thus interpret organ bacterial loads from GE values. Image to the left says ACOD+/+ - should be +/-.

<u>Response:</u> *Immunohistochemical staining correlated very well with the GE data on day 7 after infection (Fig. 3C, E). We do have the data for the first experiment of day 11 after i.t. infection, where there is still significant signal for* C. burnetii *staining, but no significant difference between genotypes (Response Fig. 8). The results fit quite well to the GE data shown in Fig. 3B.*

Response Fig. 8. *C. burnetii* staining in lung tissue on day 11 after i.t. infection.

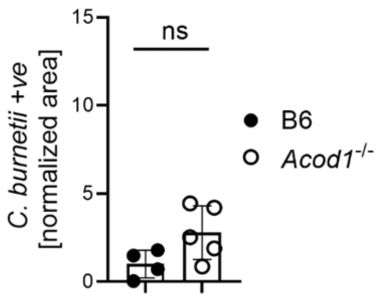

We do agree with the Reviewer that immunohistochemical validation of GE data is desirable and provides additional information. However, there are also limitations and drawbacks to this technique (see also response to Reviewer's comments to Fig. 5 below), especially the limited sensitivity and decreasing signal-to-noise ratio for samples with lower bacterial burden as judged by GE analysis. In our experience, for samples with lower bacterial burden (e.g. spleen and liver after i.t. infection; later timepoints after infection), these limitations preclude automatic image analysis of scanned sections and it was therefore not included here.
Indeed, there was a mistake in labeling the left panel of the microscopy images in Fig. 3C, in that the slide was from a B6 wild type control mouse and not an Acod1$^{+/+}$ littermate control. This has been corrected in the revised Fig. 3C.

- Since in vitro experiments suggest this is a cell-intrinsic mechanism of controlling the infection - do BMDMs represent some specific cell-intrinsic mechanism found in lung macs - but not spleen and liver macs?

Response: *We agree with the Reviewer that the results from the intratracheal infection raised the question whether the lung macrophage compartment shows a tissue-specific requirement for Acod1 (Results section, line 205-212). In fact, this question was one of the reasons why we chose to infect in addition by the i.p. route to test whether macrophages in spleen and liver may be independent of Acod1 for containment of* C. burnetii*. The results clearly show that spleen and liver macrophages also require Acod1 for efficient defense against* C. burnetii *when infection is done via the i.p. route (Fig. 3D, E), thus, the mechanism is not specific to lung macrophages, or to BMDM.*

3D - I.p. infection, even if showing that systemic infection gives a phenotype, one can hardly argue that this is more relevant than intratracheal infection since the authors say initially that the way to get Q fever is from inhalation? What happens at other time points after i.p infection, before /after day 7? Does the infection resolve?

Response: *We agree that most natural infections occur through inhalation. It was important to determine whether the Acod1$^{-/-}$ phenotype is only observed in lungs, which is not the case, as our results after i.p. infection demonstrate. In addition, another advantage of the i.p. route is that it is the more tractable model for treatment (see response below to comments for Fig. 5).*

The Reviewer raises the important question of whether infection by the i.p. route also is controlled and resolves. To address this issue, we performed additional i.p. infection experiments and assessed bacterial burden and inflammatory gene expression on day 11 after infection (comparable to the late timepoint in the intratracheal infection model). The results show that bacterial burden is strongly reduced compared to day 7 in all organs, independent of the genotype. Of note, there is still a significantly higher bacterial load in the livers and less so in the lungs of Acod1$^{-/-}$ mice (new Fig. 3F in the revised manuscript); however, the phenotype of ACOD1-deficiency is much less pronounced on day 11 than on day 7, suggesting that ACOD1 is

particularly important for control of C. burnetii *during the early phase of infection, independent of the route of infection.*

new Fig. 3F in revised manuscript:

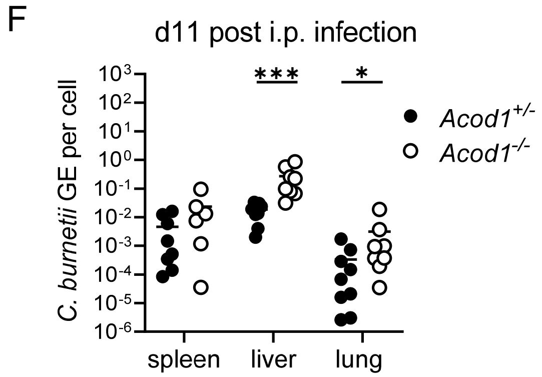

3F - there is a significant difference in liver ACOD1-/- receiving het bone-marrow, despite written in the text that there is no contribution outside the haemopoietic compartment.

<u>Response:</u> Thanks for this comment. We have now described the results of the infection experiment in the radiation chimeras in more detail, including this increase in the livers of Acod1$^{-/-}$ recipients in the results. Still, the conclusion from the experiment holds that ACOD1 is primarily required in the hematopoietic compartment.

It would be interesting to see the relative cell populations in the lungs after intratracheal infection in het/WT vs ACOD1 KO mice. Anything like what Nair et al. shows for Mtb (PMN infiltration)?

<u>Response:</u> We have not analyzed the infiltrating cell population in the organs of mice infected with C. burnetii *by flow cytometry in the experiments included in the originally submitted manuscript.*

We have included flow cytometry panels for myeloid cell infiltration in the infections done for the revision of this manuscript (d2 and d11 after i.t. infection), please see also response to comment 1) by Reviewer #1 above. We did observe a reduction in the frequency of alveolar macrophages after infection that was accompanied by a moderate increase in the percentages of monocytes and neutrophils in the lungs. There were slight differences between the genotypes (more monocytes in Acod1$^{-/-}$ on day 2, a trend for more neutrophils at day 11).

4B - why were i.p infections terminated at day 7, including bacterial burden (Fig 3)? It makes the data less informative with regards to comparing results from the two models- also for resolution.

<u>Response:</u> Following the Reviewer's suggestion, we have performed two i.p. infection experiments with analysis on day 11 (bacterial burden shown in new Fig. 3F, weight curve in new Fig. 4B, and gene expression analysis in new Fig. 4F). The results show that also in the i.p. infection model control of bacterial burden develops after day 7 (Fig. 3F), that weight loss in Acod1$^{-/-}$ mice is transient (Fig. 4B) and expression of IL-6, IFNγ, Nos2 and Gbp1 is increased on day 11 in the liver but not the spleen of Acod1$^{-/-}$ (Fig. 4F).

Furthermore, since we repeated the i.t. infection for the d11 timepoint (see Fig. 3B), we obtained additional data for the weight curve that is shown in Fig. 4A. The pooling of the body weight data from a total of six i.t. infection experiments showed a less pronounced, but still highly significant weight loss in Acod1$^{-/-}$ mice.

The weight analyses in Fig. 4A and B are now based on a quite large number of mice. In contrast, the weight curves for the radiation chimeras shown in the initially submitted manuscript were derived from comparable few mice. Although on average a larger weight loss was found in recipients of Acod1$^{-/-}$ bone marrow, the small number of mice did not allow statistical testing and the variability between the mice in one experimental group was quite large. Therefore, we decided

to remove original Fig. 4C from the revised manuscript and to delete the sentence describing these data in the Results section.

4D, E - why are the mice recovering at day 11 if not from some induced protective immune response, and what could that be - since e.g., IFNgamma is lower in the lungs and spleen at day 11 post infection when the infection resolves? Cytokine levels should preferentially be shown as protein levels - relative gene-expression cannot be used to explain biological effects. Especially since it is shown that IFNgamma can rescue ACOD1 deficiency, it would be relevant to see IFNgamma protein levels in the organs of infected mice during the 11 days of infection to see if this could explain resolution.

<u>*Response:*</u> *The question why the mice control infection and show signs of resolution at the later time point on day 11 is indeed very interesting. Given the* in vitro *observation that IFNγ can overcome the effect of Acod1-deficiency in BMM* in vitro*, the developing adaptive IFNγ response is the most likely explanation why infection resolves at day 11.*

In the revised manuscript, a second experiment for gene expression on day 11 after i.t. infection (new Fig. 4D) and two experiments for day 11 after i.p. infection (new Fig. 4F) are included. These later confirm that IFNγ, Nos2 and Gbp1 are expressed at higher levels in the livers of Acod1$^{-/-}$ mice after i.p. infection, whereas only Gbp1 was significantly increased in the lungs and spleen after i.t. infection.

The suggestion of the Reviewer to analyze IFNγ levels at the protein level is very good, and we have started to employ multiplex measurement of cytokines from organ homogenates. Indeed, new data from lung and liver homogenates of mice infected by the i.t. and i.p. route, respectively, showed increased mean IFNγ protein levels in Acod1$^{-/-}$ mice. The results from day 11 liver samples after i.p. infection are included as new Fig. 4G in the revised manuscript. Given the preliminary character of the data from the lung tissue, we do not include these in the revised manuscript, but will expand the protein-based cytokine analysis from tissue homogenates in ongoing experiments in the C. burnetii *model.*

Figure 5:

5A - using technical replicate to calculate significance is not a good practice.

<u>*Response:*</u> *We thank the Reviewer for this comment, as it prompted us to re-examine the data and correct the Figure. As stated, the experiment was done twice. In the first experiment, two mice per genotype were used to differentiate BMM. The BMM from were pooled and plated in triplicates for stimulation. The average values of the three technical replicates were calculated to obtain one biological replicate. In the second experiment, three mice per genotype were used to differentiate BMM. These were plated separately for the different stimulation conditions. Thus, in the second experiment, we had three biological replicates. In the revised Fig. 5A, data from the four biological replicates pooled from both experiments are shown. Given the clear differences between genotypes and treatment conditions, and the small number of biological replicates, we did not perform statistical testing.*

5D - should ideally do siRNA ACOD1 to demonstrate that ACOD1 is needed in MDMs.

<u>*Response:*</u> *We followed the Reviewer's suggestion and tried to knock down Acod1 mRNA levels in human MDM using siRNA, but we were not successful. In future experiments, we plan to use CRISPR approach to be able to work with ACOD1 deficient human monocytes/macrophages.*

However, it is important to keep in mind that ITA inhibits C. burnetii *replication in huMDM (Fig. 5E), whereas it does not have a significant effect in WT murine BMM. This difference between species is paralleled by a much lower capacity of huMDM to generate ITA after infection reported in the*

literature (Michelucci 2013), despite strong induction of mRNA expression (see Fig. 1B). We have performed GC-MS analysis of MDM from three human donors and found that no increase in itaconate could be detected 24 hours after infection with NMII, whereas exogenously added ITA was readily detectable at high levels as in murine BMM. The levels of succinate increased in all three donors after addition of ITA, consistent with the known inhibition of SDH; interestingly, succinate was also higher in MDM infected with NMII, which may indicate endogenous itaconate levels below the sensitivity of our GC-MS analysis. These results are now described in the Results section and are shown as Expanded View Fig. EV3.

Together, since it is reasonable to interpret that endogenous itaconate production in human MDM is suboptimal at best, the ITA treatment experiment is more relevant than the ACOD1 siRNA KD in the huMDM system.

5I/J - These data are convincing. However, the experiments should have included WT+ itaconate to check if the treatment effectively lowers the burden of WT mice - to justify the treatment suggestion. As for now, only rescue of the ACOD1 KO is shown.

Response: *Please, see also our response to Reviewer #2 general remarks above, including Response Fig. 4.*

We followed the Reviewer's suggestion and tested in the i.p. infection model whether treatment with ITA does also reduces the bacterial burden in Acod1$^{+/+}$ or Acod1$^{+/-}$ mice. These data are preliminary, as to date only one experiment was performed. As shown above in Response Fig. 4, the NMII GE in the liver, spleen and lung of ITA-treated Acod1-expressing mice were not different from those of untreated mice. The lack of a further reduction in Acod1-expressing mice suggests that endogenous production of itaconate is already sufficiently high to inhibit replication of C. burnetii. This is consistent with the results of the in vitro *infection experiments using BMM (Fig. 5B).*

For the interpretation of these results with regard to the potential of ITA as a future treatment strategy for humans, the difference in the capacity to produce itaconate between macrophages from both species needs to be considered: as shown by others, and confirmed by us in this revised manuscript, human macrophages produce at least 20-fold less itaconate than their murine counterparts (Fig. 5A, EV3). Therefore, it is likely that due to low endogenous itaconate in humans a state of functional deficiency (compared to the mice) results, that opens a window of treatment opportunity for exogenous itaconate.

Also, the experiments would be more relevant if done with the intratracheal infection model - and continued to day 11 post infection, measuring both weight over time and organ load at day 7 and 11.

Response: *We have considered this experiment because we agree that the intratracheal infection is closer to the natural infection in humans. However, the treatment with itaconate injections is not covered by our animal protocol because of the following reasons: the intratracheal infection model includes surgical preparation of the trachea; additional injection of ITA by the i.p. route was considered too stressful for the mice and carries the risk of damaging to the cervical wound sutures during manual fixation of the mice for injection. Therefore, we chose the i.p. infection route for the treatment experiments and did find efficient reduction of C.b. load in all organs, including the lungs.*

Tissue images with quantification is appreciated - and should be used more to complement GE (which is more difficult to interpret), and for all organs.

The legend (or figure) does not inform the time point of the results, but assuming it is day 7.

Response: *Indeed, the treatment experiment was terminated on day 7. We agree with the Reviewer that the use of immunohistochemistry for staining of* C. burnetii *in the tissue has certain*

advantages over the measurement of C. burnetii *DNA in homogenized tissues. These include the validation of PCR data and the provision of spatial information about the distribution of infected cells in the tissues and in relation to infiltrating leukocytes, as seen in Fig. 3C/E for lung and liver. On the other hand, there are also limitations and drawbacks of this method, especially the decreasing signal-to-noise ratio of the stainings with lower bacterial burden in the organs; in addition, variability in the signal intensity between stainings done in different experiments can be an issue. As suggested by the Reviewer, we have extended the IHC analysis to other organs and included the later timepoints after i.t. and i.p. infection. The results are in part shown here (see response to comment on Fig. 3 above) and support the conclusions of the PCR analysis for* C. burnetii *GE. However, as we found that at the later time points the signal-to-noise ratio was indeed weaker, making the automated image quantitation more prone to error, we decided not to include these data in the manuscript figures.*

Minor comments:

Line 136: Michelucci et al have shown that itaconate restrict TB growth on axenic culture, not in cells.

Response: *This is correct. Our reference to the Michelucci paper does not claim otherwise. We inserted "in axenic culture" for clarity.*

Line 137: the authors do not mention the work of Hall et al showing that IRG1 is needed to control Salmonella in a Zebra Fish model (Cell Metabolism, 2013), Nair et al (JEM, 2018) showing that IRG1 is needed to restrict TB in a mouse model, the work of Daniels et al showing that IRG1 and 4-OI are needed to control Zika virus infection in a mouse model (Immunity, 2019).

Response: *The work by Hall et al. is cited in the Discussion section of the manuscript. We include a reference to the Daniels et al. paper in the Introduction of the revised manuscript. The Nair et al. manuscript was cited in the Results section and in the Discussion for its identification of immunregulatory effects of ACOD1; we include it now also in the Introduction section, stating the increases bacterial burden linked to dysregulated neutrophil accumulation in the absence of a direct impact of ACOD1-deficiency on mycobacterial growth in macrophages.*

Figure 1a legend: Figure shows 5h and 20h post infection but 20h is not mention in the legend

Response: *Thank you very much for identifying this mistake. Both time points of 5 and 20 days after infection are now mentioned in the legend to Fig. 1A.*

Line 201 - says significantly higher load - but not where? Figures show it is in the KOs, but this should be spelled out in the text.

Response: *Corrected.*

Line 219 - "attenuated weight loss" - meaning? Sentence is nested and it is difficult in the end to interpret what is really communicated - that organ bacterial load correlates with inflammatory gene expression but not necessarily with pathology (weight loss) - or the other way around?

Response: *We split this complicated sentence in two to more clearly convey the notion that bacterial load not always correlates with inflammatory cytokine levels and clinical symptoms such as weight loss.*

Line 222-223 - GBPs and NOS are not inflammatory cytokines

Response: *This has been corrected by amending the sentence with "… and immune mediators."*

Line 366 - claim IFNgamma is high at day 7 and 11. No measurements of that?

Response: *This section of the Discussion refers to the gene expression data shown in Fig. 4C-F. Protein level measurements were performed from lung and liver homogenates only for day 11 after i.t. and i.p. infection, respectively (see response above to comment to Fig. 4D/E). IFNγ protein in liver homogenates from day 11 after i.p. infection is shown in new Fig. 4G.*

The discussion opens with saying ACOD1 is essential - this can hardly be true, since the infection is self-limiting, and no mice die? Major protective mechanism is ok.

Response: *The first sentence of the Discussion has been changed to clarify that ACOD1 is essential* in vivo *during the early phase of infection.*

Referee #3 (Comments on Novelty/Model System for Author):

Model is adequate, but strengths and weaknesses should be added to discussion. Given that these results need to be confirmed with virulent bacteria, the medical impact is medium.

Response: *In response to the Reviewers' and the Editor's comments, we have performed experiments to validate inhibition of* C. burnetii *replication by ITA using the virulent NMI strain, both in axenic culture and in infected human THP-1 macrophages (Results lines 338-347 new Fig. 5K-M). Therefore, it is reasonable to expect that the ACOD1-itaconate pathway will play a similar role during infection with virulent* C. burnetii *NMI* in vivo.

Referee #3 (Remarks for Author):

In this manuscript, Kohl et al explore the role of nutritional immunity, specifically citrate metabolism, during Coxiella infection. The mammalian enzyme ACOD1 decarboxylates cis-aconitate to itaconate (ITA); recent evidence for numerous bacterial pathogens indicates that ACOD1 and ITA restrict pathogen growth through various mechanisms. This group recently established a new mouse model for Coxiella infection, using myd88-deficient mice and avirulent Coxiella NMII. In this study, they found that ACOD1 is upregulated shortly after Coxiella infection in both the mouse as well as in vitro in mouse bone marrow-derived macrophages and primary human monocyte-derived macrophages. Coxiella infection improves in ACOD1-knockout macrophages and mice. Important points include that ACOD1 expression in hematopoietic cells is critical and that exogenous ITA inhibits Coxiella growth. Finally, ITA directly inhibits Coxiella in axenic media, though an immunomodulatory role in macrophage cannot be ruled out.

Overall, this is a very nice study, clearly and logically written with an impressive balance of in vitro and in vivo experiments. The experiments are well done, and conclusions are based on multiple lines of evidence and in general not over-interpreted. While performed with avirulent bacteria, the authors have uncovered a potentially critical component of the host immune response to Coxiella infection. While not necessarily novel for the broader field of bacterial pathogenesis, nutritional immunity in the context of Coxiella infection is relatively unexplored and is therefore of significance and high impact.

Major comments:

1) This study uses an immune-deficient mouse model (myd88 KO) as well as avirulent bacteria. While the data indicating that ITA directly inhibits Coxiella growth in axenic media helps alleviate

concerns that the ACOD1/ITA finding is an artifact of the model system, the discussion should address the strengths and weaknesses of this model, and that these findings need to be confirmed with wildtype mice and bacteria (NMI).

__Response:__ As already mentioned above and in the reply to the Editor's comments, the inhibition of bacterial replication by itaconate in axenic culture and in human macrophages has been confirmed using the virulent NMI strain (Fig. 5K-M). For future experiments with the NMI strain in mouse models in vivo*, we are currently in contact with two institutions with BSL-3 mouse facilities. However, the time required to establish the* C. burnetii*-specific SOPs and to obtain permission for these experiments is significant and beyond the time frame for this revision. These new results, and the limitation of not having investigated NMI infection in mice* in vivo*, is discussed in the revised manuscript (lines 417-423).*

Regarding the treatment of C. burnetii*-infected wild-type mice with itaconate, a test experiment was performed in mice infected i.p., which did not show a reduction of the already low* C. burnetii *bacterial burden by itaconate injection in Acod$^{+/-}$ or $^{+/+}$ mice. Please, also see the response to Reviewer #2 general remarks and comment to Fig. 5I/J for graphical depiction (Response Fig. 4) and discussion.*

As the data are preliminary, we do not include them in the revised manuscript. However, the difference in itaconate levels between mouse and humans, and its implications for treatment with itaconate, are discussed in the revised manuscript (lines 452-465).

2) For in vitro infections of mutant cells, did the authors confirm that the initial infection levels (day 0) were similar?

__Response:__ Yes, we compared GE and tdTomato-staining 4 hours after infection, after removal of non-internalized C. burnetii*. This has been described now for GE analysis in the Results section (lines 182f.) and in new Fig. 2B. In addition, Fig. 2D in the manuscript shows that the percentage of* C. burnetii *highly positive cells is not different between B6 and Acod1-/- BMM at this time. Furthermore, Fig. 5B also shows 4 hour* C. burnetii *GE data for Acod1$^{+/-}$ and Acod1$^{-/-}$ BMM.*

Minor comments

1. In many cases, the figure legends need to clarify how samples were normalized when comparing gene expression. For example, it's not clear that in figure 4 D-F that expression was first normalized to naïve/uninfected mice.

__Response:__ Thanks for this hint. The calibration procedure for gene expression analyses to samples from non-infected control mice has been described in the Methods section ("RNA isolation and gene expression analysis by qRT-PCR"). To clarify this point, we have added a statement to the legend for Fig. 4D-G.

2. Figure 5F, which shows growth in axenic media with different concentrations of ITA, should be shown as GE or CFU, which is the norm in the field. It is difficult to determine the extent of the growth defect (e.g., one log), when shown as OD.

__Response:__ We agree that quantitation of C. burnetii growth can be done by qPCR for GE or culture for CFU determination. However, "spectrophotometric measurement of culture optical density is a quick and accurate way of determining C. burnetii *replication, allowing discrimination between even minor differences in growth" (cited from Sanchez SE, Vallejo-Esquerra E, Omsland A. 2018. Use of axenic culture tools to study Coxiella burnetii. Current Protocols in Microbiology, 50, e52. Doi: 10.1002/cpmc.52). Since it allows the kinetic analysis of many samples, we used it as our starting point to determine the ITA effect in dose titration experiments on NMII (Fig. 5H) and NMI (Fig. 5K). We have validated these OD measurement results using CFU determination in*

experiments not shown in this manuscript, as well as in the experiments of delayed addition of ITA to established cultures of NMI and NMII (see Fig. 5J and Fig. 5L). Together, we are very confident that the OD measurements provide a robust measurement of the impact of ITA on replication of C. burnetii*.*

15th Nov 2022

Dear Prof. Lang,

Thank you for the submission of your revised manuscript to EMBO Molecular Medicine. I am pleased to inform you that we will be able to accept your manuscript pending the following final amendments:

1) As you will see from the referee reports below, referees #1 and #3 support publication of the manuscript, while referee #2 acknowledges the improvements of the revised manuscript but remains critical regarding the importance of ACOD1 in defense against Coxiella. Please address this point by discussion in point-by-point response, no additional discussion in the main text is required.
2) Authors: E-mail correspondence to Marta Ölke, Michael Mauermeir and Valentin Schatz could not be delivered. Please update their e-mail addresses and make sure to enter correct e-mail addresses for all authors in our submission system.
3) We note that you currently have a total of 3 first authors. Is that correct? Do you confirm equal contribution of these 3 people, able to take full responsibility for the paper and its content? While there is no limit per se to the number of first authors, 3 is rare and may not reflect as intended to the community.
4) Figures: All figures should fit one page. Currently, figures 3, 4 and 5 run over 2 pages, please correct. Also remove titles and legends form all figure files and upload individual, high-resolution files for main and EV figures. Please check "Author Guidelines" for more information:
https://www.embopress.org/page/journal/17574684/authorguide#figureformat
5) In the main manuscript file, please do the following:
- Correct/answer the track changes suggested by our data editors by working from the attached document.
- Limit keywords to max. 5.
- Remove text highlight color.
- Remove data not shown (page 12).
- Add callouts for EV Figures EV4B-D and EV5A-E.
- In M&M, provide the antibody dilutions that were used for each antibody.
- Please rename "Competing Interest" to "Disclosure Statement & Competing Interests" and move it after the "Acknowledgements". We updated our journal's competing interests policy in January 2022 and request authors to consider both actual and perceived competing interests. Please review the policy https://www.embopress.org/competing-interests and update your competing interests if necessary.
- Author contributions: Please remove it from the manuscript and specify author contributions in our submission system. CRediT has replaced the traditional author contributions section because it offers a systematic machine-readable author contributions format that allows for more effective research assessment. You are encouraged to use the free text boxes beneath each contributing author's name to add specific details on the author's contribution. More information is available in our guide to authors:
https://www.embopress.org/page/journal/17574684/authorguide#authorshipguidelines
- Correct the reference citation in the reference list. Citations should be listed in alphabetical order. Where there are more than 10 authors on a paper, 10 will be listed, followed by "et al.". Please check "Author Guidelines" for more information.
https://www.embopress.org/page/journal/17574684/authorguide#referencesformat
6) Acknowledgments: Add grant numbers in the manuscript. Please make sure that information about all sources of funding are complete in both our submission system and in the manuscript.
7) Reagent table: Please remove it and place all relevant information in the M&M section.
8) Synopsis: Every published paper now includes a 'Synopsis' to further enhance discoverability. Synopses are displayed on the journal webpage and are freely accessible to all readers. They include separate synopsis image and synopsis text.
- Synopsis image: Please provide a striking image or visual abstract as a high-resolution jpeg file 550 px-wide x (250-400)-px high to illustrate your article.
- Synopsis text: Please provide a short standfirst (maximum of 300 characters, including space) as well as 2-5 one sentence bullet points that summarise the paper as a .doc file. Please write the bullet points to summarise the key NEW findings. They should be designed to be complementary to the abstract - i.e. not repeat the same text. We encourage inclusion of key acronyms and quantitative information (maximum of 30 words / bullet point). Please use the passive voice.
- Please check your synopsis text and image before submission with your revised manuscript. Please be aware that in the proof stage minor corrections only are allowed (e.g., typos).
9) For more information: Please remove data availability information. This space should be used to list relevant web links for further consultation by our readers. Could you identify some relevant ones and provide such information as well? Some examples are patient associations, relevant databases, OMIM/proteins/genes links, author's websites, etc...
10) Source data: We encourage you to include the source data for figure panels that show essential data. Numerical data should be provided as individual .xls or .csv files (including a tab describing the data). For blots or microscopy, uncropped images should be submitted (using a zip archive if multiple images need to be supplied for one panel). Please check "Author Guidelines"

for more information. https://www.embopress.org/page/journal/17574684/authorguide#sourcedata
11) As part of the EMBO Publications transparent editorial process initiative (see our Editorial at http://embomolmed.embopress.org/content/2/9/329), EMBO Molecular Medicine will publish online a Review Process File (RPF) to accompany accepted manuscripts. This file will be published in conjunction with your paper and will include the anonymous referee reports, your point-by-point response and all pertinent correspondence relating to the manuscript. Let us know whether you agree with the publication of the RPF and as here, if you want to remove or not any figures from it prior to publication. Please note that the Authors checklist will be published at the end of the RPF.
12) Please provide a point-by-point letter INCLUDING my comments as well as the reviewer's reports and your detailed responses (as Word file).

I look forward to reading a new revised version of your manuscript as soon as possible.

Yours sincerely,

Zeljko Durdevic

Zeljko Durdevic
Editor
EMBO Molecular Medicine

***** Reviewer's comments *****

Referee #1 (Remarks for Author):

The authors responded appropriately to the reviewers' comments and criticisms, and the manuscript has further improved.

Referee #2 (Remarks for Author):

The authors have addressed all comments with new experiments including several new mouse infections, which is highly appreciated. However, the overall importance of ACOD1 is still open for discussion since all data suggest that the infection resolves even in ACOD1 KO mice and with no pathological consequences (which could be an argument for the importance of ACOD1 even if the infection resolves). This could, of course, be different for humans, and perhaps better addressed in a model of chronic Q fever if this is possible to establish e.g., with the virulent strain. The comment that ACOD1 is essential for restricting Coxiella growth in macrophages is only valid for mouse macrophages.

Referee #3 (Comments on Novelty/Model System for Author):

Given the limitations of infecting mice with virulent bacteria, the authors have now included in vitro with virulent bacteria and found similar results to avirulent bacteria.

Referee #3 (Remarks for Author):

The authors have done a nice job with the revision and my concerns have been adequately addressed.

Reviewer's comments

Referee #1 (Remarks for Author):

The authors responded appropriately to the reviewers' comments and criticisms, and the manuscript has further improved.

Authors' response: Thanks for the positive remarks.

Referee #2 (Remarks for Author):

The authors have addressed all comments with new experiments including several new mouse infections, which is highly appreciated. However, the overall importance of ACOD1 is still open for discussion since all data suggest that the infection resolves even in ACOD1 KO mice and with no pathological consequences (which could be an argument for the importance of ACOD1 even if the infection resolves). This could, of course, be different for humans, and perhaps better addressed in a model of chronic Q fever if this is possible to establish e.g., with the virulent strain. The comment that ACOD1 is essential for restricting Coxiella growth in macrophages is only valid for mouse macrophages. *Authors' response: We thank the Reviewer again for the thorough and constructive evaluation of our initial manuscript. Addressing the points raised by the Reviewer improved the manuscript and led to several new findings in preliminary results that will deserve to be pursued in ongoing work in the lab. The fact that* C. burnetii *infection in Acod1-/- mice is controlled at later timepoints, likely through the action of other IFNg-dependent mechanisms, is discussed at length in the manuscript. We also acknowledge in the Discussion that ACOD1 „… is a key factor in the early control of infection* in vivo*". Indeed, as the Reviewer points out, the use of a virulent* C. burnetii *strain for infection of mice* in vivo *will be an important future experiment, once the logistical hurdles are cleared. This is underscored by our demonstration in the revised manuscript that itaconate inhibits the replication of the virulent Nine Mile Phase I strain in axenic culture and in a human macrophage cell line. Finally, our data show that human macrophages are relatively poor producers of itaconate, thus treatment with exogenous itaconate may boost the capacity of infected humans to control* C. burnetii.

Referee #3 (Comments on Novelty/Model System for Author):
Given the limitations of infecting mice with virulent bacteria, the authors have now included in vitro with virulent bacteria and found similar results to avirulent bacteria.

Referee #3 (Remarks for Author):
The authors have done a nice job with the revision and my concerns have been adequately addressed.
Authors' response: We thank the Reviewer for the positive feedback and for suggesting the experiment with virulent C. burnetii.

We are pleased to inform you that your manuscript is accepted for publication and is now being sent to our publisher to be included in the next available issue of EMBO Molecular Medicine.

EMBO Press Author Checklist

Corresponding Author Name: Roland Lang	
Journal Submitted to: EMBO MOL MED	
Manuscript Number: EMM-2022-15931	

USEFUL LINKS FOR COMPLETING THIS FORM
The EMBO Journal - Author Guidelines
EMBO Reports - Author Guidelines
Molecular Systems Biology - Author Guidelines
EMBO Molecular Medicine - Author Guidelines

Reporting Checklist for Life Science Articles (updated January
This checklist is adapted from Materials Design Analysis Reporting (MDAR) Checklist for Authors. MDAR establishes a minimum set of requirements in transparent reporting in the life sciences (see Statement of Task: 10.31222/osf.io/9sm4x). Please follow the journal's guidelines in preparing your manuscript.
Please note that a copy of this checklist will be published alongside your article.

Abridged guidelines for figures
1. Data
The data shown in figures should satisfy the following conditions:
- the data were obtained and processed according to the field's best practice and are presented to reflect the results of the experiments in an accurate and unbiased manner.
- ideally, figure panels should include only measurements that are directly comparable to each other and obtained with the same assay.
- plots include clearly labeled error bars for independent experiments and sample sizes. Unless justified, error bars should not be shown for technical
- if n<5, the individual data points from each experiment should be plotted. Any statistical test employed should be justified.
- Source Data should be included to report the data underlying figures according to the guidelines set out in the authorship guidelines on Data

2. Captions
Each figure caption should contain the following information, for each panel where they are relevant:
- a specification of the experimental system investigated (eg cell line, species name).
- the assay(s) and method(s) used to carry out the reported observations and measurements.
- an explicit mention of the biological and chemical entity(ies) that are being measured.
- an explicit mention of the biological and chemical entity(ies) that are altered/varied/perturbed in a controlled manner.
- the exact sample size (n) for each experimental group/condition, given as a number, not a range;
- a description of the sample collection allowing the reader to understand whether the samples represent technical or biological replicates (including how many animals, litters, cultures, etc.).
- a statement of how many times the experiment shown was independently replicated in the laboratory.
- definitions of statistical methods and measures:
  - common tests, such as t-test (please specify whether paired vs. unpaired), simple χ2 tests, Wilcoxon and Mann-Whitney tests, can be unambiguously identified by name only, but more complex techniques should be described in the methods section;
  - are tests one-sided or two-sided?
  - are there adjustments for multiple comparisons?
  - exact statistical test results, e.g., P values = x but not P values < x;
  - definition of 'center values' as median or average;
  - definition of error bars as s.d. or s.e.m.

Please complete ALL of the questions below.
Select "Not Applicable" only when the requested information is not relevant for your study.

Materials

**Newly Created Materials**	**Information included in the manuscript?**	**In which section is the information available?** (Reagents and Tools Table, Materials and Methods, Figures, Data Availability Section)
New materials and reagents need to be available; do any restrictions apply?	Not Applicable	

**Antibodies**	**Information included in the manuscript?**	**In which section is the information available?** (Reagents and Tools Table, Materials and Methods, Figures, Data Availability Section)
For **antibodies** provide the following information:<br>- Commercial antibodies: RRID (if possible) or supplier name, catalogue number and or/clone number<br>- Non-commercial: RRID or citation	Yes	Materials and methods

**DNA and RNA sequences**	**Information included in the manuscript?**	**In which section is the information available?** (Reagents and Tools Table, Materials and Methods, Figures, Data Availability Section)
**Short novel DNA or RNA including primers, probes**: provide the sequences.	Yes	Materials and Methods

**Cell materials**	**Information included in the manuscript?**	**In which section is the information available?** (Reagents and Tools Table, Materials and Methods, Figures, Data Availability Section)
**Cell lines:** Provide species information, strain. Provide accession number in repository **OR** supplier name, catalog number, clone number, and/**OR** RRID.	Yes	Materials and methods
**Primary cultures:** Provide species, strain, sex of origin, genetic modification status.	Yes	Materials and methods
Report if the cell lines were recently **authenticated** (e.g., by STR profiling) and tested for mycoplasma contamination.	Not Applicable	

**Experimental animals**	**Information included in the manuscript?**	**In which section is the information available?** (Reagents and Tools Table, Materials and Methods, Figures, Data Availability Section)
**Laboratory animals or Model organisms:** Provide species, strain, sex, age, genetic modification status. Provide accession number in repository **OR** supplier name, catalog number, clone number, **OR** RRID.	Yes	Materials and Methods
**Animal observed in or captured from the field:** Provide species, sex, and age where possible**.**	Not Applicable	
Please detail **housing and husbandry conditions**.	Yes	Material and Methods

**Plants and microbes**	**Information included in the manuscript?**	**In which section is the information available?** (Reagents and Tools Table, Materials and Methods, Figures, Data Availability Section)
**Plants:** provide species and strain, ecotype and cultivar where relevant, unique accession number if available, and source (including location for collected wild specimens).	Not Applicable	
**Microbes:** provide species and strain, unique accession number if available, and source.	Yes	Materials and Methods

**Human research participants**	**Information included in the manuscript?**	**In which section is the information available?** (Reagents and Tools Table, Materials and Methods, Figures, Data Availability Section)
If collected and within the bounds of privacy constraints report on age, sex and gender or ethnicity for all study participants.	Not Applicable	

**Core facilities**	**Information included in the manuscript?**	**In which section is the information available?** (Reagents and Tools Table, Materials and Methods, Figures, Data Availability Section)
If your work benefited from core facilities, was their service mentioned in the acknowledgments section?	Yes	Materials and Methods; Acknowledgements

Design

Study protocol	Information included in the manuscript?	In which section is the information available? (Reagents and Tools Table, Materials and Methods, Figures, Data Availability Section)
If study protocol has been **pre-registered, provide DOI in the manuscript**. For clinical trials, provide the trial registration number **OR** cite DOI.	Not Applicable	
Report the **clinical trial registration number** (at ClinicalTrials.gov or equivalent), where applicable.	Not Applicable	

Laboratory protocol	Information included in the manuscript?	In which section is the information available? (Reagents and Tools Table, Materials and Methods, Figures, Data Availability Section)
Provide DOI OR other citation details if **external detailed step-by-step protocols** are available.	Not Applicable	

Experimental study design and statistics	Information included in the manuscript?	In which section is the information available? (Reagents and Tools Table, Materials and Methods, Figures, Data Availability Section)
Include a statement about **sample size** estimate even if no statistical methods were used.	Not Applicable	
Were any steps taken to minimize the effects of subjective bias when allocating animals/samples to treatment (e.g. **randomization procedure**)? If yes, have they been described?	Yes	Materials and Methods
Include a statement about **blinding** even if no blinding was done.	Yes	Materials and Methods
Describe **inclusion/exclusion criteria** if samples or animals were excluded from the analysis. Were the criteria pre-established?<br><br>If sample or data points were omitted from analysis, report if this was due to attrition or intentional exclusion and provide justification.	Yes	Materials and Methods
For every figure, are **statistical tests** justified as appropriate? Do the data meet the assumptions of the tests (e.g., normal distribution)? Describe any methods used to assess it. Is there an estimate of variation within each group of data? Is the variance similar between the groups that are being statistically compared?	Yes	Materials and Methods; Figures

Sample definition and in-laboratory replication	Information included in the manuscript?	In which section is the information available? (Reagents and Tools Table, Materials and Methods, Figures, Data Availability Section)
In the figure legends: state number of times the experiment was **replicated** in laboratory.	Yes	Figures
In the figure legends: define whether data describe **technical or biological replicates**.	Yes	Figures

Ethics

Ethics	Information included in the manuscript?	In which section is the information available? (Reagents and Tools Table, Materials and Methods, Figures, Data Availability Section)
Studies involving **human participants**: State details of **authority granting ethics approval** (IRB or equivalent committee(s), provide reference number for approval.	Yes	Materials and Methods
Studies involving **human participants**: Include a statement confirming that **informed consent** was obtained from all subjects and that the experiments conformed to the principles set out in the WMA Declaration of Helsinki and the Department of Health and Human Services Belmont Report.	Not Applicable	
Studies involving **human participants:** For publication of **patient photos**, include a statement confirming that consent to publish was obtained.	Not Applicable	
Studies involving experimental **animals**: State details of **authority granting ethics approva**l (IRB or equivalent committee(s), provide reference number for approval. Include a statement of compliance with ethical regulations.	Yes	Materials and Methods
Studies involving **specimen and field samples:** State if relevant **permits** obtained, provide details of authority approving study; if none were required, explain why.	Not Applicable	

Dual Use Research of Concern (DURC)	Information included in the manuscript?	In which section is the information available? (Reagents and Tools Table, Materials and Methods, Figures, Data Availability Section)
Could your study fall under dual use research restrictions? Please check biosecurity documents and list of **select agents and toxins** (CDC): https://www.selectagents.gov/sat/list.htm	Not Applicable	
If you used a select agent, is the security level of the lab appropriate and reported in the manuscript?	Not Applicable	
If a study is subject to dual use research of concern regulations, is the name of the **authority granting approval and reference number** for the regulatory approval provided in the manuscript?	Not Applicable	

Reporting

The MDAR framework recommends adoption of discipline-specific guidelines, established and endorsed through community initiatives. Journals have their own policy about requiring specific guidelines and recommendations to complement MDAR.

Adherence to community standards	Information included in the manuscript?	In which section is the information available? (Reagents and Tools Table, Materials and Methods, Figures, Data Availability Section)
State if relevant guidelines or checklists (e.g., **ICMJE, MIBBI, ARRIVE, PRISMA**) have been followed or provided.	Not Applicable	
For **tumor marker prognostic studies,** we recommend that you follow the **REMARK** reporting guidelines (see link list at top right). See author guidelines, under 'Reporting Guidelines'. Please confirm you have followed these guidelines.	Not Applicable	
For **phase II and III randomized controlled trials**, please refer to the **CONSORT** flow diagram (see link list at top right) and submit the CONSORT checklist (see link list at top right) with your submission. See author guidelines, under 'Reporting Guidelines'. Please confirm you have submitted this list.	Not Applicable	

Data Availability

Data availability	Information included in the manuscript?	In which section is the information available? (Reagents and Tools Table, Materials and Methods, Figures, Data Availability Section)
Have **primary datasets** been deposited according to the journal's guidelines (see 'Data Deposition' section) and the respective accession numbers provided in the Data Availability Section?	Not Applicable	
Were **human clinical and genomic datasets** deposited in a public access-controlled repository in accordance to ethical obligations to the patients and to the applicable consent agreement?	Not Applicable	
Are **computational models** that are central and integral to a study available without restrictions in a machine-readable form? Were the relevant accession numbers or links provided?	Not Applicable	
If publicly available data were reused, provide the respective **data citations in the reference list**.	Not Applicable	
